# Obstructed labor and its effect on adverse maternal and fetal outcomes in Ethiopia: A systematic review and meta-analysis

**DOI:** 10.1371/journal.pone.0275400

**Published:** 2022-09-30

**Authors:** Yordanos Gizachew Yeshitila, Beniam Daniel, Melaku Desta, Getachew Mullu Kassa

**Affiliations:** 1 School of Nursing, College of Medicine and Health Science, Arba Minch University, Arba Minch, Ethiopia; 2 Department of Midwifery, College of Medicine and Health Science, Debre Markos University, Debre Markos, Ethiopia; 3 College of Medicine and Health Science, Debre Markos University, Debre Markos, Ethiopia; Aga Khan University pakistan, PAKISTAN

## Abstract

**Background:**

Obstructed labor is one of the five major causes of maternal mortality and morbidity in developing countries. In Ethiopia, it accounts for 19.1% of maternal death. The current review aimed to assess maternal and perinatal outcomes of obstructed labor in Ethiopia.

**Methods:**

Preferred Reporting Items for Systematic Reviews and Meta-Analyses (PRISMA) guideline was followed for this systematic review and meta-analysis. A literature search was made using PubMed/MEDLINE, CINAHL, Summon country-specific search, and Cochrane Libraries’ online databases. Search terms were adverse outcome, obstructed labor, maternal outcome, fetal outcome, and Ethiopia. The Newcastle-Ottawa scale (NOS), based on a star scoring system, was used to assess the quality of the included studies. The meta-analysis was conducted using STATA 16 software. The pooled prevalence of an adverse maternal outcome, fetal outcome, and association between adverse outcome and obstructed labor was calculated using a random-effects model. Egger’s test and funnel plot were used to evaluate publication bias.

**Result:**

Eighty-seven studies were included in this review, with an overall sample size of 104259 women and 4952 newborns. The pooled incidence of maternal death was estimated to be 14.4% [14.14 (6.91–21.37). The pooled prevalence of uterine rupture and maternal near-miss was 41.18% (95% CI: 19.83, 62.54) and 30.5% [30.5 (11.40, 49.59) respectively. Other complications such as postpartum hemorrhage, sepsis, obstetric fistula, hysterectomy, bladder injury, cesarean section, and labor abnormalities were also reported. The pooled prevalence of perinatal death was 26.4% (26.4 (95% CI 15.18, 37.7). In addition, the association of obstructed labor with stillbirth, perinatal asphyxia, and meconium-stained amniotic fluid was also demonstrated.

**Conclusions:**

In Ethiopia, the incidence of perinatal and maternal mortality among pregnant women with obstructed labor was high. The rate of maternal death and maternal near miss reported in this review was higher than incidences reported from high-income and most low and middle-income countries. Uterine rupture, postpartum hemorrhage, sepsis, fistula, hysterectomy, and bladder injury were also commonly reported. To improve the health outcomes of obstructed labor, it is recommended to address the three delay models: enhancing communities’ health-seeking behavior, enhancing transportation for an obstetric emergency with different stakeholders, and strengthening the capacity of health facilities to handle obstetric emergencies.

## Introduction

Obstructed labor (OL) is when the presenting part of the fetus cannot progress through the pelvis due to obstruction that usually occurs at the pelvic brim, in the cavity, or at the outlet of the pelvis despite strong uterine contractions [1,2], where the obstruction can only be relieved by either cesarean section or other instrumental delivery (forceps, vacuum extraction or symphysiotomy) [1].

Causes of OL are due to mechanical factors, which are mainly due to abnormal feto-pelvic relationships. They often cause prolonged labor, but OL is confirmed when further progress is impossible without assistance [3]. Another significant cause of OL can be abnormal presentations that occur more frequently in multi-parous women, where the shoulders of the fetus can also hinder passage through the pelvic outlet [3,4]. In rare cases, soft tissue abnormalities can cause OL [3].

The global number of maternal deaths per 100,000 live births declined by only 2.3% between 1990 and 2015. Nonetheless, accelerated decline rates in MMR were observed from 2000 onwards. During the same period, in sub-Saharan Africa, the reduction in the MMR rate remained hampered, and most countries in the region made sluggish progress in reducing maternal mortality [5–7]. Ethiopia was among the six countries globally that contributed to more than 50% of maternal deaths worldwide in 2008 [8,9]. OL is one of the five major causes of MMR and morbidity in developing countries [10]. In Ethiopia, it accounts for 19.1% of maternal death [11].

OL causes significant perinatal mortality and morbidity in the short and long term [12]. Maternal complications due to OL include intrauterine infection, bladder and rectal injuries due to damage during labor, and uterine rupture with subsequent bleeding, shock, or even death [1]. The long-term condition after OL is the obstetric fistula, an opening that forms in the vaginal wall and communicates in the bladder or rectum or both [1,2,13]. OL accounts for about 80–90% of obstetric fistula [2,14]. Neglected Obstetric fistula is one of the causes of MMR in developing countries [15]. Mental health issues related to OL include depression, anxiety, and social consequences such as stigma, divorce, abandonment, loss of income, and loss of property [16]. Perinatal complications of neglected OL include asphyxia which leads to stillbirth [17], brain damage, or neonatal death [1,16].

The preventable causes of MMR, such as social, cultural, economic, educational, and infrastructural factors of any given country, reflect not only the adequacy of obstetric care but also the socio-economic development level of the country [18,19]. In resource-constrained areas, the poorly functioning health system cannot guarantee the availability of a cesarean section where the amplified effect of OL is observed [10,19]. Early diagnosis and treatment of OL can prevent maternal deaths and obstetric fistulas, but this requires access to emergency obstetric care, a service that most mothers in developing countries do not have [20].

The three delay model has been considered a significant factor in averting maternal complications and mortality from the onset [21]. Studies conducted in Ethiopia showed the effect of each delay on the incidence of maternal complications related to obstructed labor [22–24]. Moreover, proper use of partograph in health facilities will enable the obstetrician to diagnose prolonged or obstructed labor early and easily is recommended [25]. WHO also emphasizes the use of partograph in line with evidence-based reference. Using recordings and reviewing their observations against these references, health personnel could avoid unnecessary interventions and act on warning signs [26]. Studies in Ethiopia reported adverse complications due to obstructed labor due to non/improper use of partogrpah [23,27,28].

Studies to date show that the problem of OL and its adverse perinatal outcomes are serious and a common public health problem in Ethiopia, and uterine rupture is the leading cause of MMR associated with OL [29–33]. The prevalence of OL in Ethiopia is estimated at 20% [8], ranging between 3.3% and 34.3% in the Tigray [32] and Oromia regions [28], respectively. Incidence of adverse feto-maternal outcomes attributed to obstructed labor were reported in different studies: post-partum hemorrhage [34–36], obstetric fistula [37–39], sepsis [31,33] maternal near miss [40–42], hysterectomy [32,33] maternal mortality [42–44], perinatal asphyxia [45–48], perinatal mortality [49–51], neonatal near miss [52], non-reassuring fetal heart rate [53], and neonatal hypothermia [54].

Identifying adverse feto-maternal outcomes associated with OL is crucial for reducing morbidity and mortality by recognizing modifiable factors and informing evidence-based public health interventions. In this review, the double burden of obstructed labor was investigated, and the incidence of postpartum haemorrhage and sepsis attributed to the prevention of OL attracted our attention. Evidence-based public health interventions to reduce OL will alleviate various adverse complications that cause severe morbidities and mortalities in both the fetus and the mother. Therefore, this systematic review and meta-analysis aimed to estimate adverse maternal and perinatal outcomes of OL.

## Methods

### Protocol and registration

This systematic review and meta-analysis were conducted following the recommendation of the Preferred Reporting Items for Systematic Reviews and Meta-Analysis (PRISMA) guidelines. This review protocol is registered in the International Prospective Register of Systematic Reviews (PROSPERO) database and can be accessed at display_record.php?RecordID=CRD42020196153https://www.crd.york.ac.uk/PROSPERO/display_record.php?RecordID=CRD42020196153. The systematic review protocol is written following the PRISMA guidelines. See S1 Checklist for the completed PRISMA-P checklist.

#### Eligibility criteria

*Study design/characteristics*. Observational studies (cross-sectional, case controls, and cohort) that reported adverse fetal and maternal outcomes of OL were included in the current review. Studies that reported adverse feto-maternal outcomes due to OL among mothers or women who have recently given birth were also included. This review included studies conducted in Ethiopia and published in English.

#### Population

Studies report OL as a cause for adverse perinatal outcome/birth outcome, and studies reporting OL and associated feto-maternal outcomes.

#### Settings

Studies conducted in Ethiopia were considered. There are 10 regions and 2 chartered cities in Ethiopia.

#### Outcome of interest

The following adverse perinatal outcomes were reviewed in the current review.

**Maternal adverse outcomes**: uterine rupture, sepsis, PPH, vesicovaginal fistula (VVF), bladder rupture, wound dehiscence, anemia, perineal tear, cervical tear, hysterectomy, maternal near-miss, labor abnormalities, adverse birth outcome, and maternal death.

**Neonatal adverse outcomes**: Asphyxia, sepsis, neonatal jaundice, birth injury (cranial injury), non-reassuring fetal heart rate fetal/, meconium-stained amniotic fluid, and perinatal mortality.

#### Information sources and search strategy

A systematic search of PubMed/MEDLINE, CINAHL, summon country-specific search, and Cochrane Libraries was conducted from inception. The search strategy was developed in PubMed/MEDLINE (see S1 File) and adapted to the other bibliographic databases. The following search terms were included in the subject headings (e.g., MeSH in PubMed/MEDLINE) for each database and free-text words for the key concepts of “adverse outcome”, “obstructed labor,” “labor complication”, “maternal near-miss”, “sepsis”, “postpartum hemorrhage”, “bladder injury”, “hysterectomy”, “neonatal near miss”, “cesarean section”, “obstetric fistula”, “birth asphyxia”, “stillbirth”, “meconium-stained amniotic fluid”, “neonatal sepsis”, “neonatal hypothermia”, “non-reassuring fetal heart rate”, “perinatal mortality, and “Ethiopia.” Grey literature was searched, including Google Scholar, Open Grey, and the World Health Organization (WHO) websites. Reference lists of included studies and related reviews were also searched manually.

#### Study selection and data extraction

**Fig 1:**
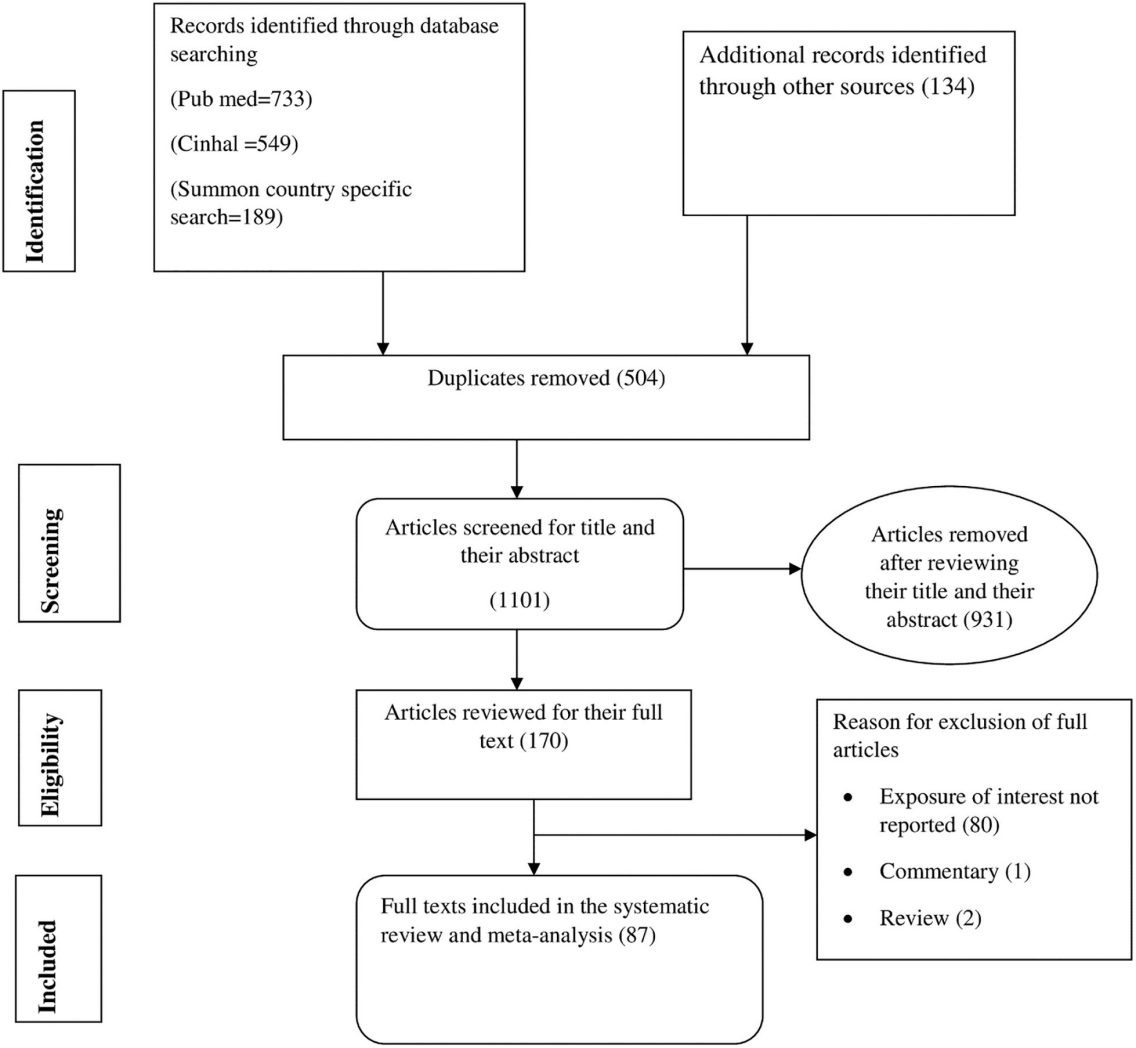
Flow diagram showing the procedures of selecting studies for meta-analysis of the effect of obstructed labor on maternal and fetal outcomes in Ethiopia, 2021.

The primary online search for the review was conducted between December 16 to March 12, 2022. We have included studies irrespective of their publication date through March 12, 2022. The citations were downloaded into the Endnote software, and duplicate articles were excluded. Two review team members (YGY, GMK) independently screened all studies identified from the literature search in two stages. In the first stage, the two reviewers (YGY, and GMK) independently screened titles and abstracts based on the eligibility criteria outlined above. Reasons for exclusion of the studies excluded from the review were documented. In the second stage, full-text versions of selected articles were downloaded/retrieved and examined in detail by the two reviewers (YGY, GMK) for eligibility. They extracted data from eligible papers identified during the full-text screening step. Any disagreements were resolved through discussion between the two authors. References of all considered articles were hand-searched to identify any relevant report missed in the search strategy. The following information were extracted using validated standard data extraction form: first author, region in which the study was conducted, year of publication, study design, sample size, outcome reported, and study population. Data were extracted independently by two authors (YGY, and GMK). In case of missing data, the corresponding and last authors of studies were communicated by email. A decision on whether to include the study in the final review was made after the authors failed to provide additional information. See (Fig 1), a flow chart showing the studies included and excluded at each stage of the study selection process.

#### Assessment of methodological quality

The Newcastle-Ottawa scale (NOS), based on a star scoring system, was used to assess the quality of the included studies [55]. The tool focuses on three components: the methodological quality of each study which is graded by five stars, the comparability of the studies, graded by two stars; and the last component of the tool assesses the outcomes and statistical analysis of the original studies, which was graded from three stars. Quality assessment was checked independently by two authors, and any disagreements were solved by discussion. Studies that received a score of 6 or above were considered high quality [56]. See (S3 File).

#### Publication bias and statistical analysis

Egger’s tests were used to assess publication bias [57]. Publication bias was declared at a p-value of less than 0.05. *I*^*2*^ test statistics were used to investigate the presence of heterogeneity across the included studies. The *I*^*2*^ test statistics of 25, 50, and 75% were declared low, moderate, and high heterogeneity, respectively, and a p-value less than 0.05 was used to declare statistically significant heterogeneity. A random-effect model was used as a method of analysis to test results with the presence of heterogeneity [58,59].

Data were extracted in Microsoft Excel and exported to STATA version 16 for further analysis. A Forest plot was used to present the combined estimate with a 95% confidence interval (CI) of the meta-analysis. Subgroup analysis was conducted for regions of the country. A meta-regression model was done based on sample size, year of publication, and quality score to identify the sources of random variations among included studies. The effect of obstructed labor on adverse maternal and perinatal morbidity and mortality was analyzed using separate categories of meta-analysis. The meta-analysis findings were presented using a forest plot and Odds Ratio (OR) with a 95% Confidence Interval (CI).

## Results

### Study selection

A total of 1605 records were retrieved through electronic database searching. Five hundred four (504) duplicate articles were removed. Records were screened using titles, abstracts, and a full article review. Consequently, we excluded 931 articles using their title and abstract review. The remaining one hundred seventy articles were assessed for eligibility, and eighty-five article were excluded. Finally, eighty-seven articles were included in this meta-analysis (Fig 1).

### Characteristics of included studies

The study included sixty-three cross-sectional, twenty-five case-control, and two cohort study designs. The largest sample size was 68,002 from the national survey [60], and the lowest sample was 27 in a study conducted in the Amhara region [61]. Overall, the review was conducted among 104259 women and 4952 newborns. All of the studies were conducted in the six regions of the country. Of these, twenty-two studies were from SNNPR, twenty-two were from the Amhara region, fifteen were from the Tigray region, twelve were from the Oromia region, six were from Addis Ababa, and three were from Harrari city administration. Additionally, seven national reviews were included (see S2 File for a detailed description).

#### Quality of the included studies

The Newcastle-Ottawa scale (NOS) was used to assess the quality of the included studies. Two studies were assessed using the NOS checklist for prospective cohort, twenty-four case studies were assessed using the NOS checklist for case-control studies, and sixty-one studies were considered the adapted version of cohort study assessment of the NOS scale. None of the studies were excluded based on the quality assessment criteria.

#### Maternal mortality due to obstructed labor

**Fig 2:**
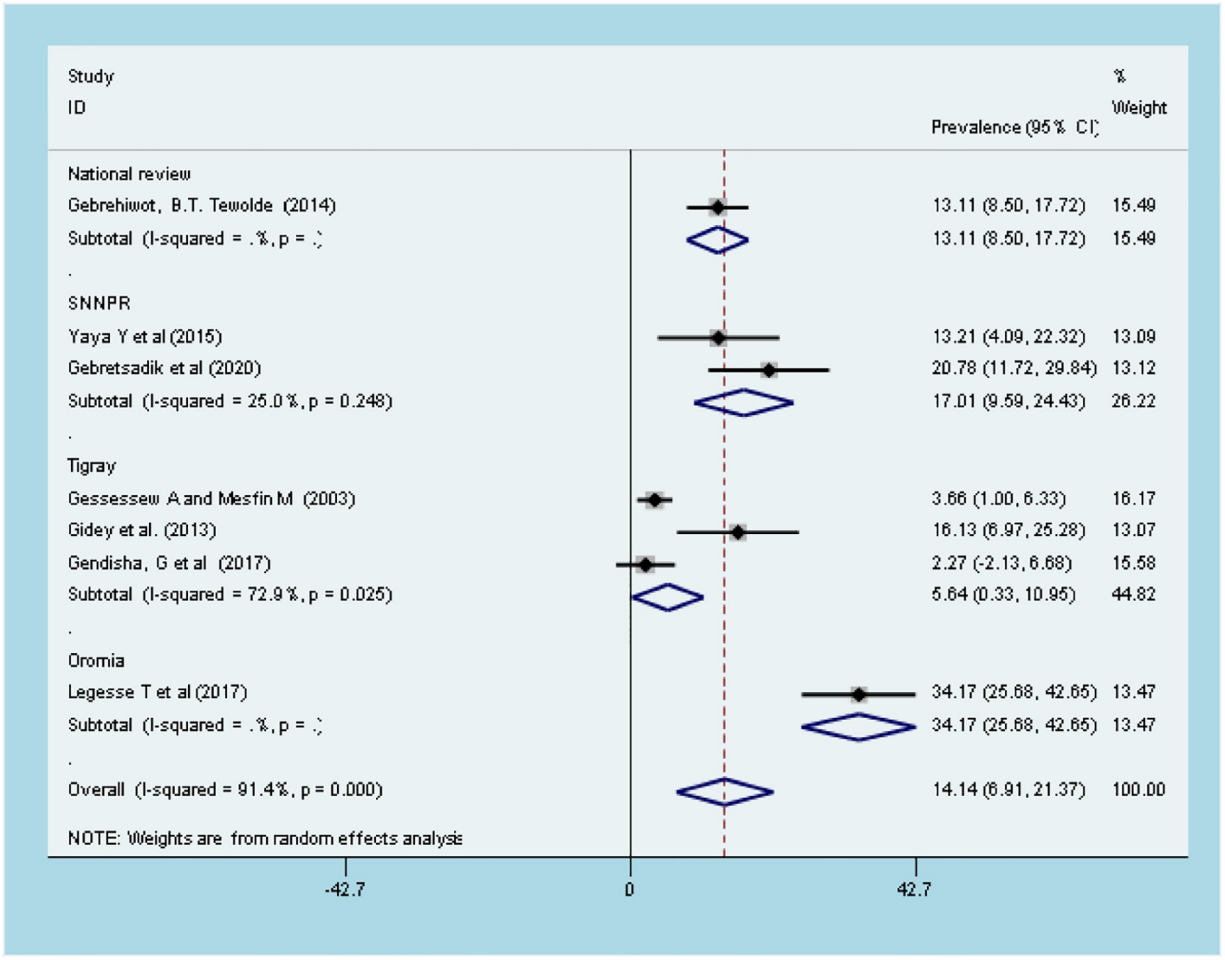
Forest plot of overall *incidence* of maternal death among obstructed labor women in Ethiopia.

Seven studies in the review reported the maternal death rate in women diagnosed with OL [31,32,42–44,62,63]. As illustrated in the forest plot, the overall rate of maternal death in women with obstructed labor was estimated to be 14.4% [14.14 (6.91–21.37), I^2^ = 91.4%, P < 0.001] (Fig 2). The test of publication bias using Egger’s test was significant, p-value > 0. 0.034. Accordingly, a univariate meta-regression was done to identify the source of publication bias. However, a non-significant heterogeneity was found due to sampling size (*p-value = 0*.*513)* and year of the study (*p-value = 0*.*23)*. According to the subgroup analysis, the highest rate of maternal death due to OL was reported from the Oromia region (34.17 (95%CI 25.68, 42.65), and the lowest rate was reported from the Tigray region (5.64 (95% CI: 0.33, 10.95) (Fig 2).

#### Severe maternal morbidity due to obstructed labor

**Fig 3:**
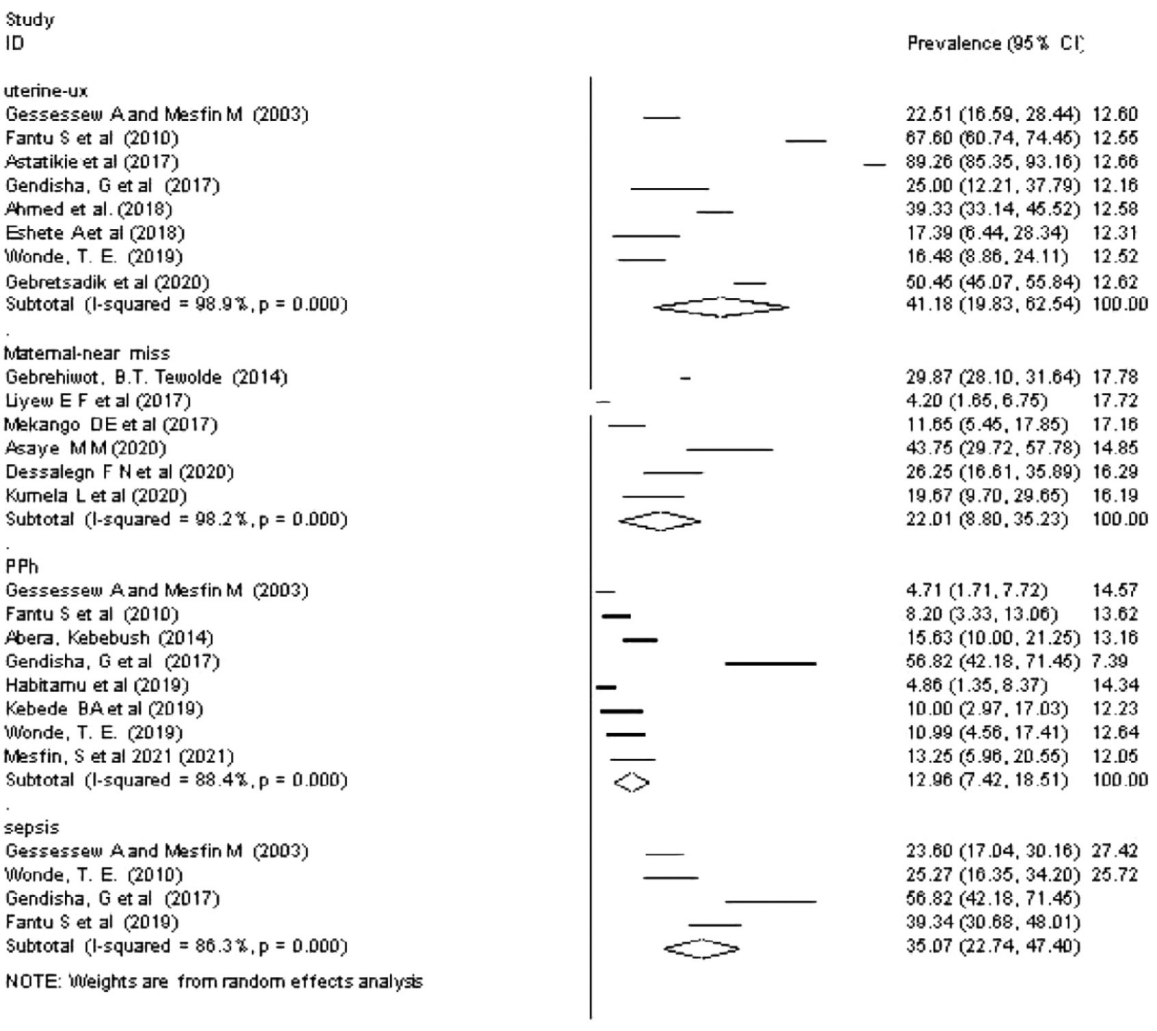
Forest plot of overall prevalence of severe maternal outcomes among obstructed labor women in Ethiopia, 2021.

*Uterine rupture due to obstructed labor*. The meta-analysis of 8 studies [29–33,64–66] showed the pooled prevalence of uterine rupture due to OL in Ethiopia to be 41.18% (95% CI: 19.83, 62.54). A random-effects model of analysis was used due to a significant heterogeneity (*I*^*2*^ = 98.9%, *p-value < 0*.*01*) (Fig 3). There was no publication bias based on the Eggers test (*p-value = 0*.*556*). However, a univariate meta-regression analysis revealed a non-significant heterogeneity due to the sample size (*p-value = 0*.*487*), year of publication (*p-value =* 0.940), quality score (*p-value =* 0.402), and study design.

(*P-value =* 0.093). The subgroup analysis revealed that the highest prevalence of uterine rupture due to OL occurred in the Oromia region (67.6% (95% CI: 60.74, 74.45), and the lowest was in the Tigray region (20.8% (95% CI: 16.41, 25.12) (Table 1).

**Table 1:** Subgroup analysis of adverse maternal morbidity due to obstructed labor by region in Ethiopia.

Adverse maternal mortality	Region	Number of studies	Prevalence [95%CI]	I2	P-value
Uterine rupture	Amhara	2	64.35 (15.43, 113.23)	99.4%	<0.001
	SNNPR	2	34.28 (1.89, 66.67)	96.5%	<0.001
	Tigray	3	20.8(16.41, 25.19)	0.0%	0.34
	Oromia	1	67.6 (60.74, 74.45)	-	-
Maternal near miss	Addis Ababa	1	4.20 (1.65,6.75)	-	-
	Amhara	1	43.75 (29.7,57.78)	-	-
	National	1	29.87(28.1,31.64)	-	-
	Tigray	1	11.65(5.45,17.85)	-	-
	Oromia	3	41.65(-0.81.1,84.11)	98.9%	<0.001
Postpartum hemorrhage	Harari	1	13.25(5.96, 20.55)		
	Addis Ababa	1	15.63 (10, 21.25)	-	-
	Amhara	1	4.86 (1.35, 8.37)	-	-
	Oromia	1	8.2 (3.33, 13.06)	-	-
	Tigray	3	22.3 (3.58, 41.02)	95.8%	<0.001
	SNNPR	1	10 (2.97, 17.03)	-	-
Sepsis	Oromia	1	25.27 (16.35,34.2)	-	-
	Tigray	3	38.9 (21.75, 56.02)	90%	<0.001
Obstetric Fistula	Oromia	2	29.93(-21.37, 81.23)	98.4%	<0.001
	Tigray	3	8.078 (5.123, 11.027)	0.0%	0.374
	Amhara	1	0.364 (0.008, 0.72)	-	-
	National	2	1.662 (-0.774, 4.098)	99.6%	<0.001

*Maternal near-miss due to obstructed labor*. Seven studies reported the prevalence of maternal near-miss due to OL and the pooled prevalence of maternal near-miss was estimated to be 30.5% [30.5 (11.40, 49.59), I^2^ = 99.3%, p < 0.001] (Fig 3) [40–42,67–70]. Egger’s test showed a non-significant publication bias, (p-value > 0.830). The subgroup analysis revealed that the highest prevalence of maternal near-miss due to obstructed labor occurred in the Amhara region (43.7% (95% CI: 29.7, 57.78) and the lowest was in Addis Ababa city administration (4.20% (95% CI: 1.65, 6.75) (Table 1).

*Postpartum hemorrhage due to obstructed labor*. A pooled prevalence of 12.96% [12.96 (7.42, 18.51), I^2^ = 88.4%, p < 0.01] was estimated for postpartum hemorrhage due to OL from the eight studies [29,31–36,71] that reported the outcome (Fig 3). There was a significant publication bias, (p-value > 0.003) and univariate meta-regression analysis revealed a non-significant heterogeneity due to the sample size (*p-value = 0*.*092)*, year of publication (*p-value =* 0.300), and quality score (*p-value =* 0.319). The subgroup analysis revealed that the highest prevalence of postpartum hemorrhage due to OL occurred in the Tigray region (22.3% (95% CI: 3.58, 41.03), and the lowest was in the Amhara region (4.86% (95% CI: 1.35, 8.37) (Table 1).

#### Maternal sepsis due to obstructed labor

Four studies [29,31–33] reported the prevalence of maternal sepsis due to OL, and the pooled prevalence of maternal sepsis was estimated to be 35.07% [35.07 (22.74, 47.40), I^2^ = 86.3%, p < 0.001] while there was no significant publication bias (p-value > 0.139) (Fig 3). The subgroup analysis revealed that the highest prevalence of postpartum hemorrhage due to OL occurred in the Tigray region (38.9 (95% CI: 21.75, 56.02), and the lowest was in the Oromia region (25.27 (95% CI: 16.35, 34.2) (Table 1).

#### Maternal morbidity due to obstructed labor

**Fig 4:**
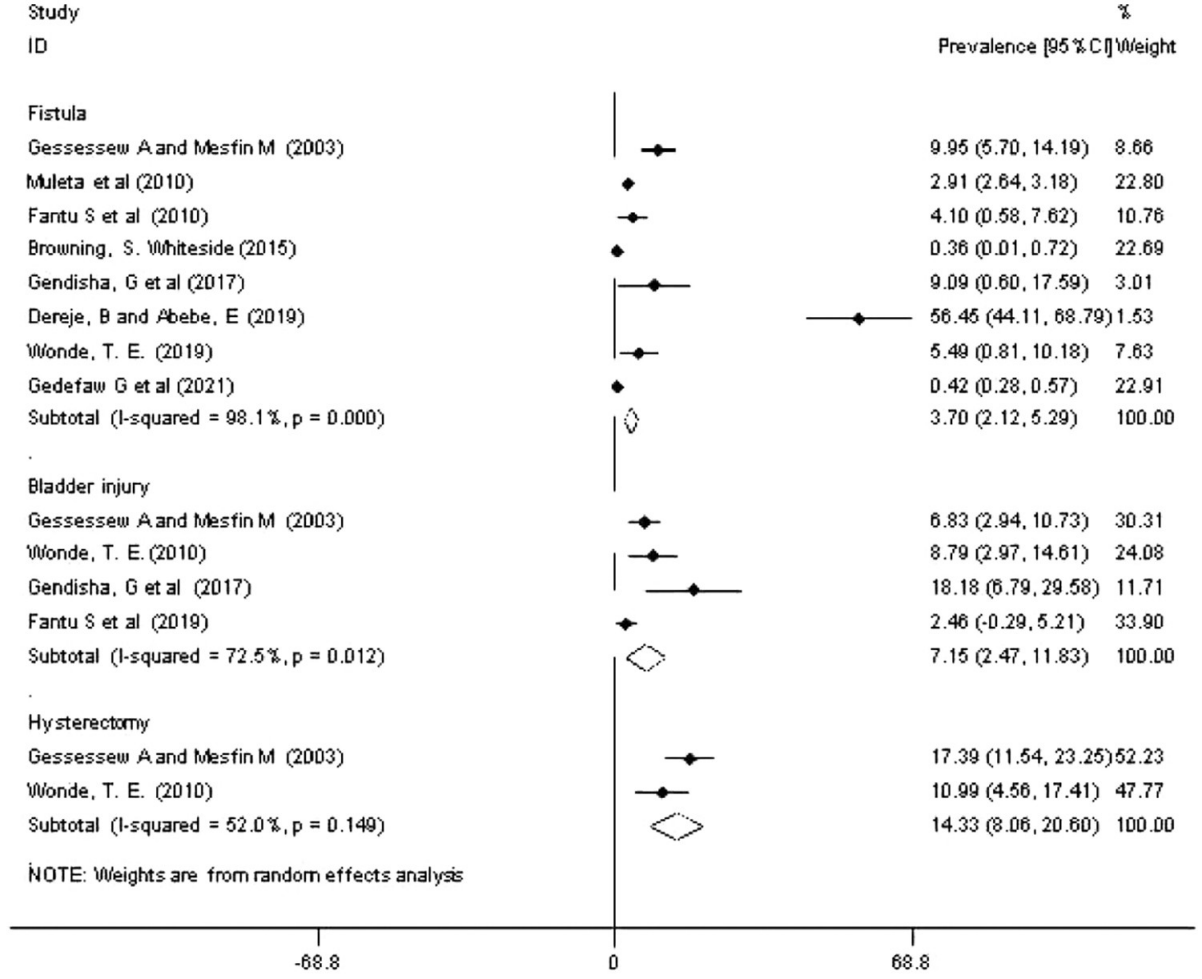
Forest plot of overall prevalence of maternal morbidities outcomes among obstructed labor women in Ethiopia, 2021.

As demonstrated in the forest plot, the overall rate of fistula in women with OL was estimated to be 3.7% [3.7 (2.12, 5.29), I^2^ = 98.1%, P < 0.001] (Fig 4). Egger’s test was nonsignificant (p-value > 0.176). The sub-group analysis showed that the highest rate of the fistula is reported in the Oromia region (29.9%); and the lowest rate was observed in the Amhara region (0.36) (Table 1). In addition, the pooled prevalence of bladder injury among women with OL was estimated to be 7% [7.15 (2.47–11.83), I^2^ = 72.5%, p < 0.012] (Fig 4). The prevalence of bladder injury was reported in two regions; the highest rate was observed in the Tigray region, 18.2% [31], and 8.8% in the Oromia region [33]. As shown in the forest plot, the overall rate of hysterectomy in women with OL was 14.3% [14.3 (8.06–20.60), I^2^ = 52%, p < 0.149] (Fig 4).

**Fig 5:**
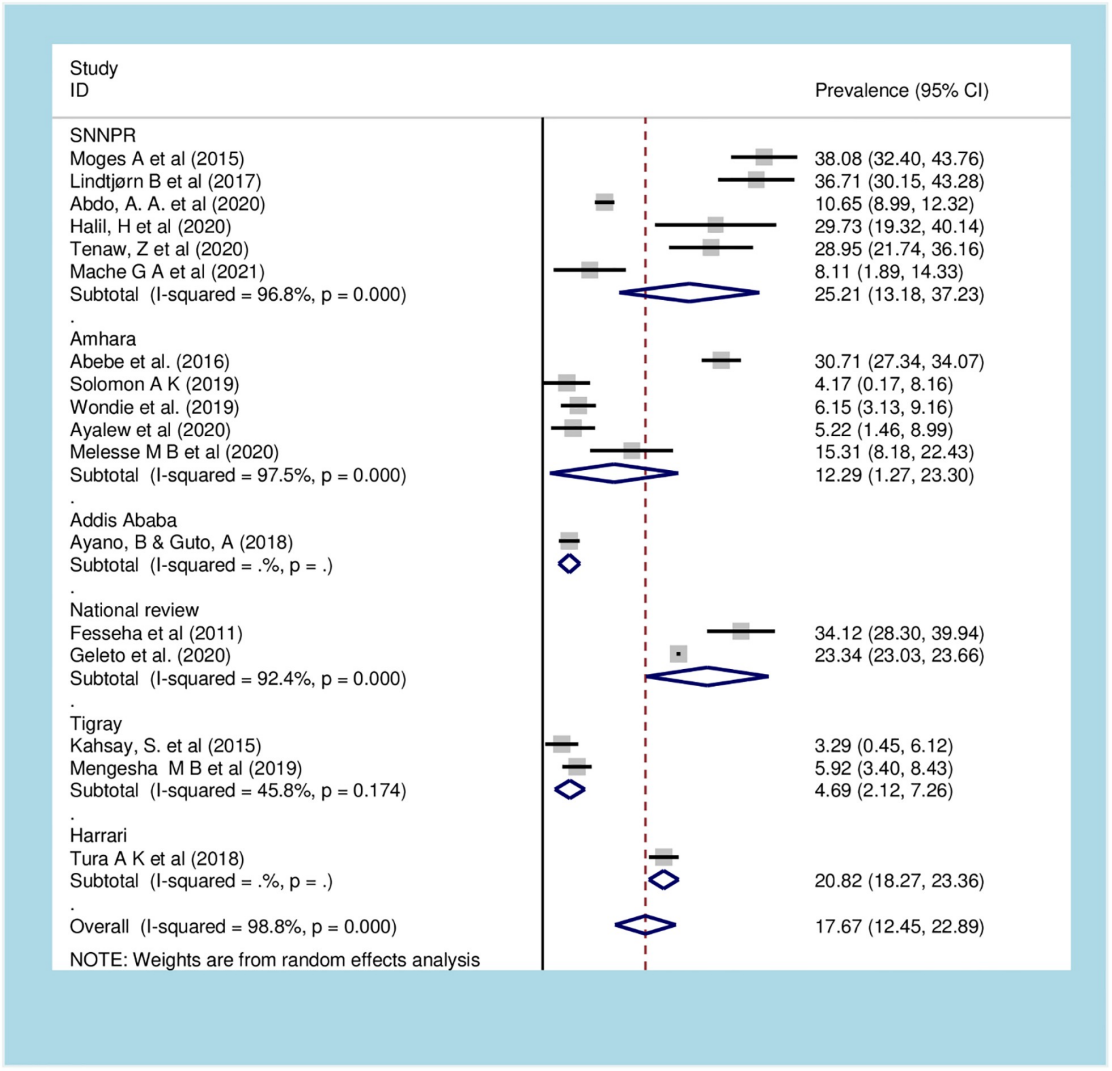
Forest plot of subgroup analysis of cesarean section among obstructed labor women in Ethiopia, 2021.

*Cesarean section due to obstructed labor*. The meta-analysis of 17 [53,60,72–86] studies showed the pooled prevalence of cesarean section due to OL in Ethiopia to be 17.67% (95% CI: 12.45, 22.89). A random-effects model of analysis was used due to a significant heterogeneity (*I*^*2*^ = 98.8%, *p-value < 0*.*01*) (Fig 5). There was no publication bias (*p-value = 0*.*064)* and a univariate meta-regression revealed a non-significant heterogeneity due to the sample size (*p-value = 0*.*346*), and study designs (*p-value 0*.*625)*, but significant heterogeneity due year of publication (*p-value =* 0.050). Subgroup analysis revealed that the highest prevalence of cesarean section due to OL occurred in national representative reviews (28.32% (95% CI: 17.80, 38.85) and the lowest was in Addis Ababa city administration (4.6% (95% CI: 2.8, 6.39) (Fig 5).

A single study in the SNNPR region reported a 17% incidence of labor abnormalities in a woman with obstructed labor [87].

#### Perinatal morbidity and mortality due to obstructed labor

**Fig 6:**
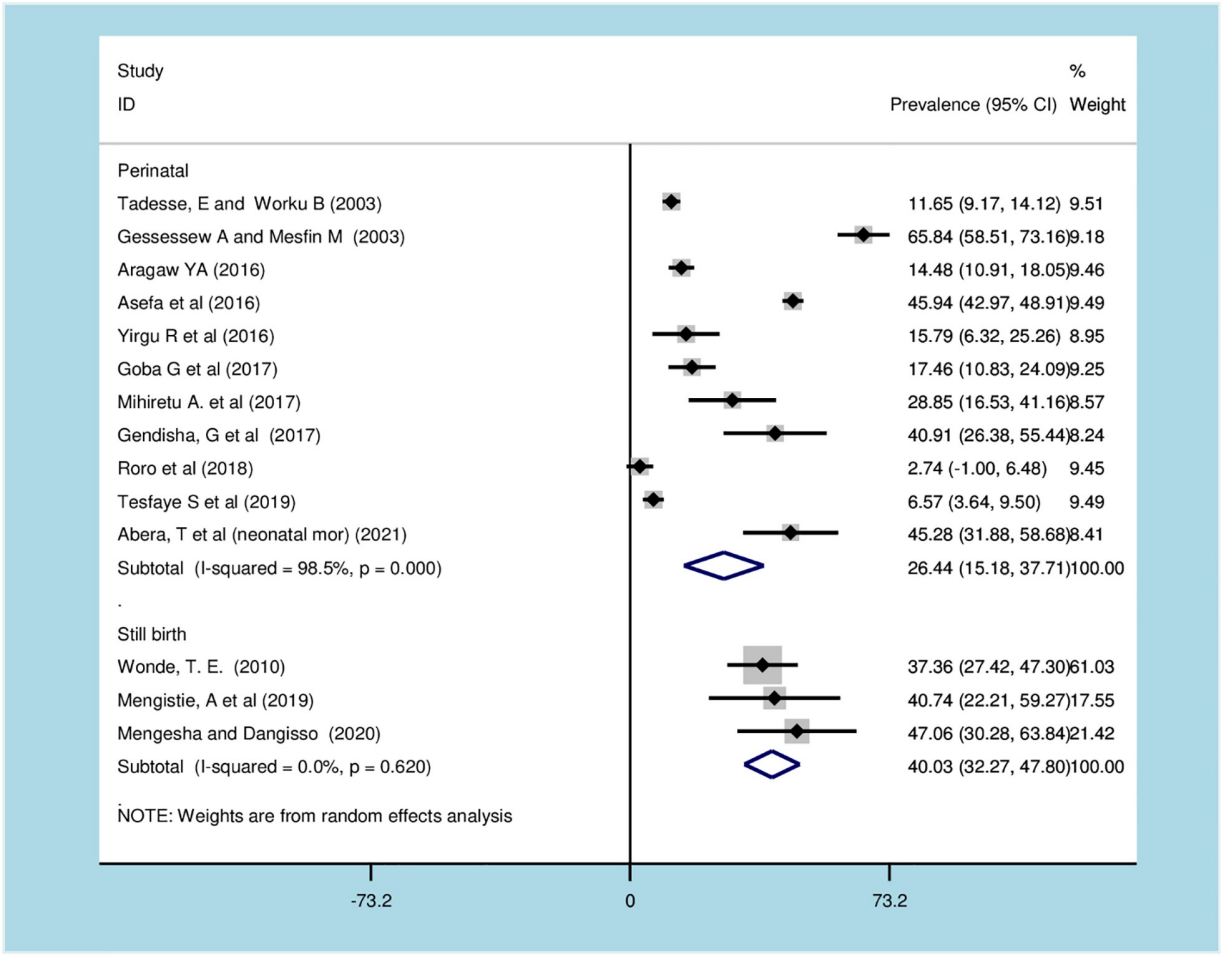
Forest plot of overall prevalence of perinatal death among obstructed labor women in Ethiopia, 2021.

Fourteen studies [31,32,49–51,88–93] reported an incidence of perinatal death in women with obstructed labor, and the forest plot in Fig 6 shows the rate of perinatal death among women with obstructed labor was 26.4% (26.4 (95% CI 15.18, 37.7), I^2^ = 95.6%, p <0.001) (Fig 6). Three studies [33,61,94] separately reported incidence of stillbirth among women with OL, 47% (95% CI: 30.3, 63.84), 37.36% (95%CI: 27.42, 47.3), and 40.74% (95% CI: 22.2, 59.2) from SNNPR, Oromia, and Amhara regions respectively. Egger’s test was not statistically significant, p-value = 0.237, and subgroup analysis showed that the highest perinatal death rate due to OL occurred in the Tigray region (Table 2).

**Table 2:** Subgroup analysis of adverse perinatal outcome due to obstructed labor by region in Ethiopia.

Adverse perinatal outcome	Region	Number of studies	Prevalence [95%CI]	I^2^	P-value
Perinatal death	Amhara	1	15.79 (6.3, 25.25)	-	-
	SNNPR	2	16.88 (-4.89, 38.6)	91.6%	<0.001
	Tigray	3	41.39 (7.454, 75.3)	97.8%	<0.001
	Oromia	4	26.78 (3.88, 49.67)	99.2%	<0.001
	Addis Ababa	1	11.6 (9.17, 14.1)	-	-

Few individual studies reported the incidence of adverse neonatal outcomes among women with OL. A study from Eastern Ethiopia stated a 10.1% incidence of neonatal hypothermia among newborns born from women with OL [54]. A study from the Amhara region reported a 12.4% prevalence of neonatal sepsis among women with obstructed labor [95]. Similarly, two studies conducted in the Amhara region demonstrated a prevalence of neonatal near miss and non-reassuring fetal heart rate, 19.15% and 20%, respectively [52,53].

#### Association between obstructed labor and adverse maternal and fetal outcomes

**Fig 7:**
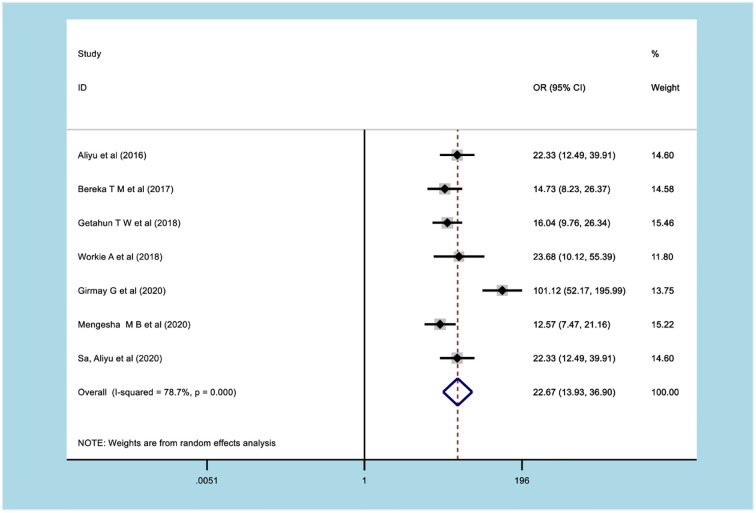
Forest plot of association of obstructed labor with uterine rupture.

The meta-analysis of 8 studies [96–102] demonstrated that OL significantly predicted uterine rupture. The odds of uterine rupture were 22.67 times higher among women with OL (OR: 22.67; 95% CI: 12.49, 39.91) (Fig 7).

**Fig 8:**
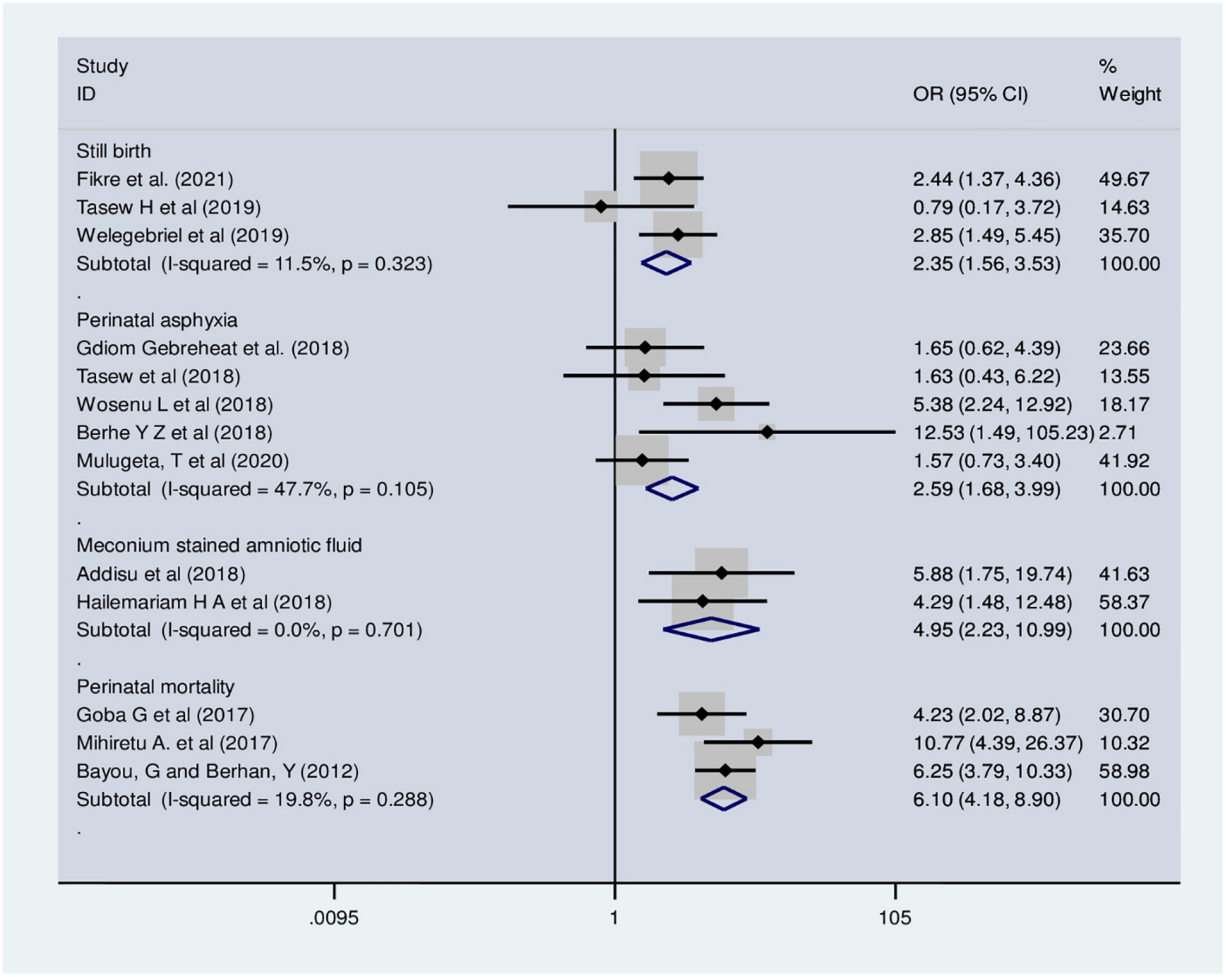
Forest plot of association of obstructed labor with uterine rupture.

Thirteen studies demonstrated the association between OL and adverse perinatal outcomes. The odds of stillbirth were 2.35 times higher among women with OL (OR: 2.35; 95% CI: 1.56, 3.53) (Fig 8). The meta-analysis of 5 studies demonstrated that OL was a significant predictor of perinatal asphyxia. The odds of perinatal asphyxia were 2.59 times higher among women with OL (OR: 2.59; 95% CI: 1.68, 4). The odds of perinatal mortality were 6.1 times higher among women with OL (OR: 6.1; 95% CI: 4.18, 8.9). Similarly, the odds of meconium-stained amniotic fluid were 4.95 times higher among women with OL (OR: 4.95; 95% CI: 2.23, 11).

## Discussion

This study estimated the pooled prevalence of maternal and perinatal morbidity and mortality due to OL among Ethiopian women. In the current review, OL accounted for 14% of maternal deaths in Ethiopia. The high incidence of maternal death in OL can be attributed to a ruptured uterus or puerperal infection [103], as observed in this review. This finding is similar to a study conducted in Nigeria and Pakistan that reported a maternal death rate of 7.7% [104] and 10% [10], respectively. However, the rate of maternal death from this review is much higher than in studies conducted in Nigeria, 6.03–6.07% [105,106], Uganda,1.21% [107], Sudan, 4.8%, Pakistan, 1.19–2.06% [108,109], and study conducted in developing countries[18]. The possible justification for the discrepancies could be the better provision of services such as antenatal care in maternal health service utilization. Tessema et al. [110] reported that Ethiopia uses the lowest recommended ANC among other African countries. Antenatal care allows early identification and management of obstetric complications [111]. The discrepancy could also be explained by the differences in the quality of maternal and neonatal health care services provided.

In Ethiopia, OL accounts for 30% of maternal near-miss cases. This proportion is higher than the studies reported from Namibia, 9.3% [112], and central Uganda,11.2% [113]. The difference in the presented proportions could be due to the inconstant use of criteria for maternal near-miss in most African countries [114].

In this review, higher proportions of uterine rupture, postpartum hemorrhage, 41.8% and 12.96%, respectively attributed to obstructed labor are reported. The highest incidence of uterine rupture and postpartum haemorrhage in OL could be explained by the prolonged duration of labor, which usually precedes OL. It results in maternal dehydration, infection, ketosis, and exhaustion [103,115]. As a result, a decrease in strength and frequency of contractions contributes to the rupture of the uterus and associated bleeding. In addition, sustained contractions of the uterus in myometrial energy depletion and hypoxia are likely to lead to myometrial edema and necrosis, contributing to uterine rupture [103].

The rate of postpartum hemorrhage reported in this review is in line with findings from East African countries [106,116], South Asian countries [108,117], and a study conducted among low and middle-income countries [118]. However, lower than the results reported from Pakistan [109,119], India [12], and Bangladesh [120]. The discrepancy could be due to the high proportion of maternal deaths secondary to obstructed labor in the Asian region [121].

The incidence of uterine rupture due to OL demonstrated in this review is similar to findings from Nigeria [104] and [122]. But, higher than results from India [117], Nepal [123], Bangladesh [120], and Pakistan [108,109,122]. The higher incidence of uterine rupture could be explained by the lack of advancement in technologies or availability of resource, which could help prevent the complications from OL. In low resource settings, the major cause of morbidity and mortality from OL is believed to be constrained resources [10].

Our review demonstrated the high incidence of sepsis, 35% in women with OL. The prolonged rupture of the membranes from a prolonged state of labor accompanied by an open cervix impairs natural, mechanical barriers to ascending infection from the vagina, which results in intrapartum infection [17,124]. This is in agreement with findings from Nigeria [104,105]. The rate of sepsis from this review is much higher than in studies from Sudan [116], Uganda [107], Pakistan [108,109,119], and India [117,125]. In addition, low- and middle-income countries reported a low incidence of sepsis [118]. The inconsistency might be due to the difference in the adequacy of facilities and equipped staff in managing women with OL. In addition, frequency of vaginal examinations during the prolonged stages of labor could contribute to the highest incidence of intrapartum infection.

Incidence of obstetric fistula (3.7%), bladder injury (7.1%), and hysterectomy (14%) were maternal complications reported by the current review. Damage to the vaginal and bladder tissues confined between the fetal head and the mother’s pubic symphysis during prolonged OL results in vesicovaginal fistulas [126,127]. The ruptured uterus and associated damage from the OL result in the observed rate of hysterectomy. The incidence of the fistula is mainly reported in developing countries: Nigeria [104], East African countries [107,116,128], India [117], and Pakistan [109,119,129]. In developed nations, fistula is a rare problem [121]. The incidence of bladder injury due to OL was stated in different research findings [12,109,117,119,120,128]. Similarly, hysterectomy due to obstructed labor was reported in some studies [119,120,130].

We have found that 17.67% of women with OL had a cesarean section. The incidence of cesarean section in a woman with OL is because the OL can be avoided by operative delivery of the fetus [20]. The reported incidence of cesarean section is much lower than in studies conducted in different regions worldwide. The incidence ranged from 82% to 95.23% [107,108,117,120,131]. The large discrepancies could be due to facilities’ availability and adequacy of emergency cesarean sections. In addition, the type of obstruction, whether relative or absolute OL, might determine the indication of cesarean section.

In this review, we have reported an incidence of perinatal death of 26.4% due to OL. The adverse perinatal outcomes attributed to OL are due to the preceding prolonged labor, which results in intrapartum infection. Moreover, the interruption of the placental exchange by strong and continued uterine contraction and retraction [115] often results in the death of the fetus either before delivery or shortly afterward from a combination of asphyxia and infection. These findings are in line with studies from different African countries [104,105,116,132], Bangladesh [120], and India [117,125]. However, there was a lower incidence of perinatal death due to OL in Uganda (14%). The observed variation in the incidence could be due to the difference in the severity of maternal morbidity, which affected the neonatal outcome. In addition, the difference in readiness for the management of immediate neonatal complications could be the reason.

We have also investigated the association between OL and perinatal outcomes. The odds of perinatal asphyxia were 2.6 more likely to occur among babies born from mothers with obstructed labor. The possible explanation could be the prolonged interruption of the placental exchange secondary to OL. This finding is supported by studies from Nigeria [133] and Pakistan [108]. Likewise, the odds of meconium-stained amniotic fluid were almost five times higher among women with OL. This finding is in line with a study from Nigeria [134]. Similar studies also reported the rate of meconium-stained amniotic fluid in women with obstructed labor [108,117,120].

### Strength and limitations of the study

The review included several articles to investigate the effect of OL on maternal and fetal outcomes. In this review, we have included studies that show the causal relationship. By doing so, the review provides valuable evidence that may contribute to the filling the gaps in research related to adverse outcomes associated with OL and informing practice and policy about the most frequent complications of OL. The review also included studies conducted in the community setting. However, the results of this review should be interpreted with some limitations. There is a high level of heterogeneity among the included studies that may have led to insufficient power to detect statistically significant associations, which should be considered while using the results of the review. In addition, outcomes such as hysterectomy, the incidence of labor abnormalities, neonatal hypothermia, neonatal sepsis, neonatal near miss, and stillbirth are represented by a small number of studies that may not be representative to conclude. The studies were conducted only in the country’s six regions, which may reduce its representativeness.

## Conclusion and recommendations

The systematic review and meta-analysis demonstrated that obstructed labor is associated with adverse maternal and neonatal morbidities and mortalities. In addition, further severe morbidities such as uterine rupture, postpartum hemorrhage, maternal sepsis, maternal near-miss, obstetric fistula, high incidence of cesarean section, bladder injury, hysterectomy, labor complications, perinatal asphyxia, meconium-stained amniotic fluid, neonatal sepsis, and non-reassuring fetal heart rate were also reported.

The advocacy for preventing the catastrophic effect of obstructed labor should be collaborative interactions amongst a range of stakeholders, and interest groups working on promoting maternal and child health. Policies and programs targeting maternal and child health should emphasize the different levels and places where the complications happen, and typical example could be advocating the three-delay model, which has been proven to avert adverse obstetric outcomes at different stages. Addressing the three delay models: enhancing communities’ health-seeking behavior, improving transportation for obstetric emergencies with other stakeholders, and strengthening the capacity of health facilities to hand over obstetric emergencies. Moreover, at health facilities enhancing the use of Partograph, a low-cost tool for monitoring labor progress and reducing the occurrence of prolonged/obstructed labor to provide an early warning system with evidence-based references is recommended. In resource-constrained areas, Partograph has practical benefits in terms of ease of use, time resourcefulness, continuity of care, and educational assistance. These positive aspects may contribute to improving maternal and fetal outcomes. Furthermore, comprehensive and targeted community education about obstructed labour combined with a wide range of activities at health facilities, including welcoming system of maternal health services, and accessible emergency obstetric services, are fundamental in reducing the incidence and complications associated with obstetric labor.

## Supporting information

S1 Checklist PRISMA 2020 flow diagram for new systematic reviews, which included searches of databases, registers, and other sources on Obstructed labor and its effect on adverse maternal and fetal outcomes in Ethiopia: A systematic review and meta-analysis. (DOCX)

S1 File The search strategy used for the systematic and meta-analysis on obstructed labour and its effect on adverse maternal and fetal outcomes in Ethiopia. (DOC)

S2 File Characteristics of included studies. (DOC)

S3 File Quality assessment of studies included in this systematic review and meta-analysis on the effect of obstructed labour on perinatal outcome in Ethiopia: A systematic review and meta-analysis. (DOC)

## Abbreviations

MMR: Maternal Mortality Ratio
NOS: Newcastle-Ottawa scale
OL: Obstructed labor
PRISMA-P: Preferred Reporting Items for Systematic Reviews and Meta-Analysis
SNNPR: Southern Nations, Nationalities, and People’s Region

## References

[1] Dolea C, AbouZahr CJWHO. Global burden of obstructed labour in the year 2000. 2003:1–17.

[2] Yeshitila YG, Desta M, Kebede A. Obstructed labor and its association with adverse feto-maternal outcome in Ethiopia: a protocol for a systematic review and meta-analysis. Syst Rev. 2021;10(1):57. Epub 2021/02/18. doi: 10.1186/s13643-021-01611-x . 33593436 PMC7887789

[3] Kwast BEJM. Obstructed labour: its contribution to maternal mortality. 1992;8(1):3–7. 10.1016/s0266-6138(05)80060-9 1598090

[4] Wells J CJTAR. The new “obstetrical dilemma”: stunting, obesity and the risk of obstructed labour. 2017;300(4):716–31. 10.1002/ar.23540 28297186

[5] World Health Organization W. Maternal Mortality [fact sheet]. 2019 [updated 16 February 2018; cited 2019 10/03/2019].

[6] Alkema L, Chou D, Hogan D, Zhang S, Moller A-B, Gemmill A, et al. Global, regional, and national levels and trends in maternal mortality between 1990 and 2015, with scenario-based projections to 2030: a systematic analysis by the UN Maternal Mortality Estimation Inter-Agency Group. The Lancet. 2016;387(10017):462–74. doi: 10.1016/s0140-6736(15)00838-7 26584737 PMC5515236

[7] WHO U, UNFPA, World Bank Group and the United Nations Population Division. Trends in Maternal Mortality: 1990 to 2015. Geneva27, Swizerlnad: 2015.

[8] Fedral Minstry Of Health Ethiopia (FMOH). Federal Ministry Of Health: Maternal Death Surveillance and Response Technical Guideline. 2014.

[9] Hogan MC, Foreman KJ, Naghavi M, Ahn SY, Wang M, Makela SM, et al. Maternal mortality for 181 countries, 1980–2008: a systematic analysis of progress towards Millennium Development Goal 5. Lancet. 2010;375(9726):1609–23. Epub 2010/04/13. doi: 10.1016/S0140-6736(10)60518-1 . 20382417

[10] Khooharo Y, Majeed T, Khawaja MA, Majeed N, Majeed N, Malik MN, et al. Even in 21st century still obstructed labor remains life threatening condition. 2012;18(3):279.

[11] Kuruvilla S, Bustreo F, Kuo T, Mishra CK, Taylor K, Fogstad H, et al. The Global strategy for women’s, children’s and adolescents’ health (2016–2030): a roadmap based on evidence and country experience. Bulletin of the World Health Organization. 2016;94(5):398–400. Epub 2016/05/02. doi: 10.2471/BLT.16.170431 . 27147772 PMC4850541

[12] Gupta R, Porwal S. Obstructed labour: incidence, cause and outcome international Journal of biological and medical research 2012.

[13] Bimbola O K JEJoRiMS. Obstructed labour: The main cause of vesico-vaginal fistula–Review of literature. 2013;1(1):1–7.

[14] Bailey PE, Andualem W, Brun M, Freedman L, Gbangbade S, Kante M, et al. Institutional maternal and perinatal deaths: a review of 40 low and middle income countries. BMC pregnancy and childbirth. 2017;17(1):295. doi: 10.1186/s12884-017-1479-1 28882128 PMC5590194

[15] Jungari S, Chauhan BG JHiL-rS. Obstetric fistula in Assam, India: a neglected cause of maternal morbidities and mortality. 2014;2:4663.

[16] Roa L, Caddell L, Ganyaglo G, Tripathi V, Huda N, Romanzi L, et al. Toward a complete estimate of physical and psychosocial morbidity from prolonged obstructed labour: a modelling study based on clinician survey. 2020;5(7):e002520. doi: 10.1136/bmjgh-2020-002520 J BMJ Global Health. 32636314 PMC7342481

[17] Neilson J, Lavender T, Quenby S, Wray S JBmb. Obstructed labour: reducing maternal death and disability during pregnancy. 2003;67(1):191–204. 10.1093/bmb/ldg018 14711764

[18] Lewis G. Maternal mortality in the developing world: why do mothers really die? Obstet Med. 2008;1(1):2–6. Epub 2008/09/01. doi: 10.1258/om.2008.080019 . 27630738 PMC5010106

[19] Monjok E, Okokon IB, Opiah MM, Ingwu JA, Ekabua JE, Essien EJ JAJoRH. Obstructed labour in resource-poor settings: the need for revival of symphysiotomy in Nigeria. 2012;16(3). 23437503

[20] Alkire BC, Vincent JR, Burns CT, Metzler IS, Farmer PE, Meara JG JPo. Obstructed labor and caesarean delivery: the cost and benefit of surgical intervention. 2012;7(4):e34595. doi: 10.1371/journal.pone.0034595 22558089 PMC3338803

[21] Pacagnella RC, Cecatti JG, Parpinelli MA, Sousa MH, Haddad SM, Costa ML, et al. Delays in receiving obstetric care and poor maternal outcomes: results from a national multicentre cross-sectional study. BMC pregnancy and childbirth. 2014;14(1):159. doi: 10.1186/1471-2393-14-159 24886330 PMC4016777

[22] Ahmed Y, Solomon l, Girma A. Prevalence and Management Outcome of Obstructed Labor among Mothers Who Gave Birth Between January, 2013 and December, 2015 in Metu Karl Referal Hospital, Ilu Ababora Zone, South West Ethiopia. EC GYNAECOLOGY. 2017.

[23] Dile M, Demelash H, Meseret L, Abebe F, Adefris M, Goshu YA, et al. Determinants of obstructed labor among women attending intrapartum care in Amhara Region, Northwest Ethiopia: A hospital-based unmatched case–control study. 2020;16:1745506520949727. doi: 10.1177/1745506520949727 . 32842920 PMC7453442

[24] Gudina A. Magnitude of Obstructed Labor and Associated Risk Factors among Mothers Come for Delivery Service in Adama Hospital Medical College, Oromia Regional State, Central Ethiopia. Journal of Gynecology and Obstetrics. 2016;4:12. doi: 10.11648/j.jgo.20160403.11

[25] KIP JP. THE PREVALENCE OF OBSTRUCTED LABOUR AMONG PREGNANT WOMEN AT A SELECTED HOSPITAL, WEST WOLLEGA, ETHIOPIA 2013.

[26] World health Organization W. Monitoring childbirth in a new era for maternal health 2020 [cited 2022 4/44/2022]. https://www.who.int/news/item/15-12-2020-monitoring-childbirth-in-a-new-era-for-maternal-health.

[27] Abdo R, Halil H JJWsHC. Magnitude and Factors Associated With Obstructed Labor among Women Delivered at Halaba Kulito Primary Hospital, Halaba Special District, Southern Ethiopia. 2019;8(453):2167–0420.

[28] Wube TT, Demissie B, Assen Z, Gelaw K, Fite RO. Magnitude of Obstructed Labor and Associated Factors Among Women Who Delivered at Public Hospitals of Western Harerghe Zone, Oromia, Ethiopia. Science publishing Group. 2018.

[29] Fantu S, Segni H, Alemseged F. Incidence, causes and outcome of obstructed labor in jimma university specialized hospital. Ethiopian journal of health sciences. 2010;20(3):145–51. doi: 10.4314/ejhs.v20i3.69443 . 22434973 PMC3275845

[30] Gebretsadik A, Hagos H, Tefera K. Outcome of uterine rupture and associated factors in Yirgalem general and teaching hospital, southern Ethiopia: a cross-sectional study. BMC Pregnancy & Childbirth. 2020;20(1):1–7. doi: 10.1186/s12884-020-02950-8 . Language: English. Entry Date: In Process. Revision Date: 20201219. Publication Type: journal article. Journal Subset: Biomedical. 32345255 PMC7189562

[31] Gendisha G, Gudayu T, Kondale M, Wakgari N, Shimbre M. Feto-maternal outcomes in obstructed labor in Suhul General Hospital, North Ethiopia. 2017:77–84.

[32] Gessessew A, Mesfin M. Obstructed labour in adigrat zonal hospital, Tigray Region, Ethiopia. Ethiopian Journal of Health Development. 2003;17(3):175–80.

[33] Wonde TE, Mihretie A. Maternofetal outcomes of obstructed labor among women who gave birth at general hospital in Ethiopia. BMC research notes. 2019;12(1):128. Epub 2019/03/15. doi: 10.1186/s13104-019-4165-8 . 30867028 PMC6416895

[34] Abera K. Magnitude, Associated Factors and Maternal Outcome of Postpartum Hemorrhage at Black Lion Specialised Hospital From Jan. 1, 2009 To Dec. 30, 2013 GC: Addis Ababa University; 2014.

[35] Habitamu D, Goshu YA, Zeleke LB. The magnitude and associated factors of postpartum hemorrhage among mothers who delivered at Debre Tabor general hospital 2018. BMC research notes. 2019;12(1):618. Epub 2019/09/25. doi: 10.1186/s13104-019-4646-9 . 31547856 PMC6757371

[36] Kebede BA, Abdo RA, Anshebo AA, Gebremariam BM. Prevalence and predictors of primary postpartum hemorrhage: An implication for designing effective intervention at selected hospitals, Southern Ethiopia. PLoS One. 2019;14(10):e0224579. Epub 2019/11/02. doi: 10.1371/journal.pone.0224579 . 31671143 PMC6822730

[37] Muleta M, Rasmussen S, Kiserud T. Obstetric fistula in 14,928 Ethiopian women. Acta Obstet Gynecol Scand. 2010;89(7):945–51. Epub 2010/04/20. doi: 10.3109/00016341003801698 . 20397760

[38] Gedefaw G, Wondmieneh A, Getie A, Bimerew M, Demis A JIJoWsH. Estimating the Prevalence and Risk Factors of Obstetric Fistula in Ethiopia: Results from Demographic and Health Survey. 2021;13:683. doi: 10.2147/IJWH.S306221 34262358 PMC8273908

[39] Dereje B, Abebe E. Assessment of Obstetric Fistula and Factors Associated among Women admitted to Jimma Medical Center, South West Ethiopia. International Journal of Healthcare Systems Engineering. 2019;1(003).

[40] Asaye MM. Proportion of Maternal Near-Miss and Its Determinants among Northwest Ethiopian Women: A Cross-Sectional Study. International journal of reproductive medicine. 2020;2020:1–9. doi: 10.1155/2020/5257431 32258094 PMC7104127

[41] Dessalegn FN, Astawesegn FH, Hankalo NC. Factors Associated with Maternal Near Miss among Women Admitted in West Arsi Zone Public Hospitals, Ethiopia: Unmatched Case-Control Study. Journal of pregnancy. 2020;2020:6029160. Epub 2020/07/23. doi: 10.1155/2020/6029160 Department of Public Health, College of Medicine and Health Sciences, Maddawalabo University; FH and NC are lecturers in the School of Public Health, College of Medicine and Health Sciences, Hawassa University. 32695514 PMC7352151

[42] Gebrehiwot Y, Tewolde BT. Improving maternity care in Ethiopia through facility based review of maternal deaths and near misses. International journal of gynecology and obstetrics. 2014;127:S29–S34. doi: 10.1016/j.ijgo.2014.08.003 25261109

[43] Gebretsadik A, Tarekegne Z, Teshome M. Retrospective review of maternal deaths in Hawassa Comprehensive Specialised Hospital, in Southern Ethiopia. Journal of obstetrics and gynaecology: the journal of the Institute of Obstetrics and Gynaecology. 2020;40(5):659–65. Epub 2019/09/13. doi: 10.1080/01443615.2019.1648398 . 31512545

[44] Yaya Y, Data T, Lindtjørn B. Maternal mortality in rural south Ethiopia: outcomes of community-based birth registration by health extension workers. PLoS One. 2015;10(3):e0119321. Epub 2015/03/24. doi: 10.1371/journal.pone.0119321 . 25799229 PMC4370399

[45] Abdo RA, Halil HM, Kebede BA, Anshebo AA, Gejo NG. Prevalence and contributing factors of birth asphyxia among the neonates delivered at Nigist Eleni Mohammed memorial teaching hospital, Southern Ethiopia: a cross-sectional study. BMC Pregnancy & Childbirth. 2019;19(1):1–7. doi: 10.1186/s12884-019-2696-6 . Language: English. Entry Date: In Process. Revision Date: 20200611. Publication Type: journal article. Journal Subset: Biomedical. 31888542 PMC6937931

[46] Gebreheat G, Tsegay T, Kiros D, Teame H, Etsay N, Welu G, et al. Prevalence and Associated Factors of Perinatal Asphyxia among Neonates in General Hospitals of Tigray, Ethiopia, 2018. BioMed Research International. 2018:1–7. doi: 10.1155/2018/5351010 . Language: English. Entry Date: 20181105. Revision Date: 20191114. Publication Type: Article. 30515406 PMC6236773

[47] Tasew H, Zemicheal M, Teklay G, Mariye T, Ayele E. Risk factors of birth asphyxia among newborns in public hospitals of Central Zone, Tigray, Ethiopia 2018. BMC research notes. 2018;11(1):496. Epub 2018/07/22. doi: 10.1186/s13104-018-3611-3 . 30029614 PMC6053756

[48] Wayessa Z, Belachew T, Joseph J JJoM, Health R. Birth asphyxia and associated factors among newborns delivered in Jimma zone public hospitals, Southwest Ethiopia: A cross-sectional study. 2018;6(2):1289–95.

[49] Aragaw Y. Perinatal mortality and associated factor in Jimma university specialized hospital, South West Ethiopia. Gynecologie, obstetrique & fertilite. 2016;6(409):2161–0932.

[50] Asefa D, Akessa G, Araya F, Amenu D, Girma W. Pattern of perinatal mortality among deliveries at Jimma University teaching hospital, South-West Ethiopia. Women’s Health. 2016;6(5):2.

[51] Goba GK, Tsegay H, Gebregergs GB, Mitiku M, Kim KA, Alemayehu M. A facility-based study of factors associated with perinatal mortality in Tigray, northern Ethiopia. International Journal of Gynecology & Obstetrics. 2018;141(1):113–9. doi: 10.1002/ijgo.12438 128376346. Language: English. Entry Date: 20180413. Revision Date: 20190603. Publication Type: journal article. 29318613

[52] Belay H, Limenih S, Wassie T, Ambie M. Neonatal Near Miss and Its Associated Factors at Injibara General Hospital, Awi Zone, Northwest Ethiopia, 2019. Exploratory Research and Hypothesis in Medicine. 2019.

[53] Kassahun EA, Aweke AM, Getu AA, Gela GB, Limenih SK, Mekonnen ME, et al. Proportion and Associated Factors of Nonreassuring Fetal Heart Rate Patterns in Finote Selam Primary Hospital, North West Ethiopia. BioMed Research International. 2020:1–7. doi: 10.1155/2020/6948972 . Language: English. Entry Date: 20201005. Revision Date: 20201005. Publication Type: Article. PMC7525310 33015176

[54] Alebachew Bayih W, Assefa N, Dheresa M, Minuye B, Demis S. Neonatal hypothermia and associated factors within six hours of delivery in eastern part of Ethiopia: a cross-sectional study. BMC Pediatr. 2019;19(1):N.PAG-N.PAG. doi: 10.1186/s12887-019-1632-2 . Language: English. Entry Date: In Process. Revision Date: 20201015. Publication Type: journal article. Journal Subset: Biomedical. 31340772 PMC6651976

[55] Wells G, Shea B, O’Connell D, Peterson j, Welch V, Losos M, et al. The Newcastle–Ottawa Scale (NOS) for Assessing the Quality of Non-Randomized Studies in Meta-Analysis. 2000;.

[56] Farsad-Naeimi A, Asjodi F, Omidian M, Askari M, Nouri M, Pizarro AB, et al. Sugar consumption, sugar sweetened beverages and Attention Deficit Hyperactivity Disorder: A systematic review and meta-analysis. Complementary Therapies in Medicine. 2020;53:102512. doi: 10.1016/j.ctim.2020.102512 33066852

[57] Lin L, Chu H. Quantifying publication bias in meta-analysis. Biometrics. 2018;74(3):785–94. Epub 2017/11/15. doi: 10.1111/biom.12817 . 29141096 PMC5953768

[58] Higgins JPT, Thompson SG, Spiegelhalter DJ. A re-evaluation of random-effects meta-analysis. J R Stat Soc Ser A Stat Soc. 2009;172(1):137–59. doi: 10.1111/j.1467-985X.2008.00552.x . 19381330 PMC2667312

[59] Riley RD, Higgins JPT, Deeks JJ. Interpretation of random effects meta-analyses. 2011;342:d549. doi: 10.1136/bmj.d549 J BMJ. 21310794

[60] Geleto A, Chojenta C, Taddele T, Loxton D. Association between maternal mortality and caesarean section in Ethiopia: a national cross-sectional study. BMC Pregnancy & Childbirth. 2020;20(1):N.PAG-N.PAG. doi: 10.1186/s12884-020-03276-1 . Language: English. Entry Date: In Process. Revision Date: 20210711. Publication Type: journal article. Journal Subset: Biomedical. 33023536 PMC7539527

[61] Mengistie A, Asemahagn M, Tamirat K. Prevalence of stillbirth and associated Factors among immediate post-partum mothers at Felegehiwot comprehensive specialized hospital, northwest Ethiopia: An institution based cross-sectional study2019.

[62] Gidey G, Bayray A, Gebrehiwot H. Patterns of maternal mortality and associated factors; a case-control study at public hospitals in Tigray region, Ethiopia, 2012. International Journal of Pharmaceutical Sciences Research. 2013;4(5):1918.

[63] Legesse T, Abdulahi M, Dirar A. Trends and causes of maternal mortality in Jimma University Specialized Hospital, southwest Ethiopia: a matched case-control study. Int J Womens Health. 2017;9:307–13. Epub 2017/05/13. doi: 10.2147/IJWH.S123455 . 28496370 PMC5422567

[64] Ahmed DM, Mengistu TS, Endalamaw AG. Incidence and factors associated with outcomes of uterine rupture among women delivered at Felegehiwot referral hospital, Bahir Dar, Ethiopia: cross sectional study. BMC Pregnancy & Childbirth. 2018;18(1):N.PAG-N.PAG. doi: 10.1186/s12884-018-2083-8 . Language: English. Entry Date: In Process. Revision Date: 20190308. Publication Type: journal article. Journal Subset: Biomedical. 30445936 PMC6240227

[65] Astatikie G, Limenih MA, Kebede M. Maternal and fetal outcomes of uterine rupture and factors associated with maternal death secondary to uterine rupture. BMC Pregnancy & Childbirth. 2017;17:1–9. doi: 10.1186/s12884-017-1302-z . Language: English. Entry Date: 20180727. Revision Date: 20190603. Publication Type: journal article. 28403833 PMC5389173

[66] Eshete A, Mekonnen S, Getachew F JEMJ. Prevalence and factors associated with rupture of gravid uterus and feto-maternal outcome: a one-year retrospective cohort study. 2018;56(1).

[67] Kumela L, Tilahun T, Kifle D. Determinants of Maternal Near Miss in Western Ethiopia. Ethiop J Health Sci. 2020;30(2):161–8. Epub 2020/03/14. doi: 10.4314/ejhs.v30i2.3 . 32165805 PMC7060379

[68] Liyew EF, Yalew AW, Afework MF, Essén B. Incidence and causes of maternal near-miss in selected hospitals of Addis Ababa, Ethiopia. PloS one. 2017;12(6):e0179013–e. doi: 10.1371/journal.pone.0179013 28586355 PMC5460898

[69] Mekango DE, Alemayehu M, Gebregergs GB, Medhanyie AA, Goba G. Determinants of maternal near miss among women in public hospital maternity wards in Northern Ethiopia: A facility based case-control study. PloS one. 2017;12(9):e0183886–e. doi: 10.1371/journal.pone.0183886 28886034 PMC5590854

[70] Abdulrazaq B, Getahun M, Mohammed A, Kedir S, Nurahmed N, Abrha Y, et al. Determinant factors of maternal near miss in selected health facilities of Berak Woreda, Oromia national regional state, Ethiopia. 2020.

[71] Mesfin S, Dheresa M, Fage SG, Tura AK JIjowsh. Assessment of Postpartum Hemorrhage in a University Hospital in Eastern Ethiopia: A Cross-Sectional Study. 2021;13:663. 10.2147/IJWH.S300143 PMC8273907 34262356

[72] Abdo AA, Hinderaker SG, Tekle AG, Lindtjørn B. Caesarean section rates analysed using Robson’s 10-Group Classification System: a cross-sectional study at a tertiary hospital in Ethiopia. BMJ Open. 2020;10(10):e039098. Epub 2020/10/30. doi: 10.1136/bmjopen-2020-039098 . 33115900 PMC7594350

[73] Ayalew M, Mengistie B, Dheressa M, Demis A JJoMH. Magnitude of Cesarean Section Delivery and Its Associated Factors Among Mothers Who Gave Birth at Public Hospitals in Northern Ethiopia: Institution-Based Cross-Sectional Study. 2020;13:1563. doi: 10.2147/JMDH.S277747 33235456 PMC7678705

[74] Ayano B, Guto A. Indications and outcomes of emergency caesarean section at St Paul’s hospital medical college, Addis Ababa, Ethiopia 2017:(afoul month retrospective cohort study). Gynecol Reprod Health. 2018;2(5):1–12.

[75] Abebe FE, Worku Gebeyehu A, Negasi Kidane A, Aynalem Eyassu G. Factors leading to cesarean section delivery at Felegehiwot referral hospital, Northwest Ethiopia: a retrospective record review. Reproductive health. 2016:1–7. doi: 10.1186/s12978-015-0114-8 . Language: English. Entry Date: 20160122. Revision Date: 20160127. Publication Type: Article. 26792611 PMC4721205

[76] Fesseha N, Getachew A, Hiluf M, Gebrehiwot Y, Bailey P. A national review of cesarean delivery in Ethiopia. International journal of gynaecology and obstetrics: the official organ of the International Federation of Gynaecology and Obstetrics. 2011;115(1):106–11. Epub 2011/08/30. doi: 10.1016/j.ijgo.2011.07.011 . 21872239

[77] Mache GA, Halil HM, Abdo RA. The Rate, Indications and Contributing Factors of Cesarean Delivery in Southern Nation Nationalities and People’s Region, Ethiopia. Journal of Midwifery & Reproductive Health. 2021;9(1):2541–7. doi: 10.22038/jmrh.2020.48476.1596 . Language: English. Entry Date: 20210222. Revision Date: 20210222. Publication Type: Article.

[78] Melesse MB, Geremew AB, Abebe SM. High prevalence of caesarean birth among mothers delivered at health facilities in Bahir Dar city, Amhara region, Ethiopia. A comparative study. PLoS One. 2020;15(4):e0231631. Epub 2020/04/17. doi: 10.1371/journal.pone.0231631 . 32299089 PMC7162673

[79] Mengesha MB, Adhanu HH, Weldegeorges DA, Assefa NE, Werid WM, Weldemariam MG, et al. Maternal and fetal outcomes of cesarean delivery and factors associated with its unfavorable management outcomes; in Ayder Specialized Comprehensive Hospital, Mekelle, Tigray, Ethiopia, 2017. BMC research notes. 2019;12(1):650. Epub 2019/10/09. doi: 10.1186/s13104-019-4690-5 . 31590693 PMC6781415

[80] Moges A, Ademe B, Akessa G JAM. Prevalence and outcome of caesarean section in Attat Hospital, Gurage Zone, SNNPR, Ethiopia. 2015;7(4):8.

[81] Tura AK, Pijpers O, de Man M, Cleveringa M, Koopmans I, Gure T, et al. Analysis of caesarean sections using Robson 10-group classification system in a university hospital in eastern Ethiopia: a cross-sectional study. BMJ Open. 2018;8(4):e020520. Epub 2018/04/07. doi: 10.1136/bmjopen-2017-020520 . 29622577 PMC5892782

[82] Wondie AG, Zeleke AA, Yenus H, Tessema GA. Cesarean delivery among women who gave birth in Dessie town hospitals, Northeast Ethiopia. PLoS One. 2019;14(5):e0216344. Epub 2019/05/07. doi: 10.1371/journal.pone.0216344 . 31059526 PMC6502338

[83] Halil H, Abdo R, Helill S, Kedir R. Predictors of Cesarean Section among Women Delivered at Durame General Hospital, Southern Ethiopia. 2020.

[84] Kahsay S, Berhe G., Gebremariam A, Birhane B. Determinants of Caesarean Deliveries and its Major Indications in Adigrat Hospital, Northern Ethiopia: A Case Control Study. Epidemiology. 2015;5(3).

[85] Tenaw Z, Yohannes Z, Siyoum M, Mekonnene S, Bekele G, Astatkie A, et al. Prevalence, indications and associated factors of cesarean section delivery at public hospitals in Wolayta Zone Southern, Ethiopia. 2020.

[86] Lindtjørn B, Mitiku D, Zidda Z, Yaya Y. Reducing Maternal Deaths in Ethiopia: Results of an Intervention Programme in Southwest Ethiopia. PLoS One. 2017;12(1):e0169304. Epub 2017/01/04. doi: 10.1371/journal.pone.0169304 . 28046036 PMC5207510

[87] Abraham W, Berhan Y. Predictors of labor abnormalities in university hospital: unmatched case control study. BMC pregnancy and childbirth. 2014;14:256. Epub 2014/08/05. doi: 10.1186/1471-2393-14-256 . 25086729 PMC4129102

[88] Roro EM, Sisay MM, Sibley LM. Determinants of perinatal mortality among cohorts of pregnant women in three districts of North Showa zone, Oromia Region, Ethiopia: Community based nested case control study. BMC Public Health. 2018;18(1):N.PAG-N.PAG. doi: 10.1186/s12889-018-5757-2 . Language: English. Entry Date: In Process. Revision Date: 20190912. Publication Type: journal article. 30021557 PMC6052561

[89] Tadesse E W B. Perinatal mortality audit at Tikure Anbessa Teaching Hospital, Addis Ababa, Ethiopia: 1995 to 1996. Malawi Med J. 2003;15(3):102–4. 27528975 PMC3346030

[90] Tesfaye S, Gebru Z, Mamo M, Getahun F, Boti N. Determinants of Perinatal Mortality in Arba Minch General Hospital, Gamo Zone, Southern Ethiopia. Ethiopian Journal of Reproductive Health. 2019;11(4):7-.

[91] Yirgu R, Molla M, Sibley L, Gebremariam A. Perinatal Mortality Magnitude, Determinants and Causes in West Gojam: Population-Based Nested Case-Control Study. PLoS One. 2016;11(7):e0159390. Epub 2016/07/29. doi: 10.1371/journal.pone.0159390 . 27467696 PMC4965173

[92] Abera T, Bayisa L, Bekele T, Dessalegn M, Mulisa D, Gamtessa LC. Neonatal Mortality and Its Associated Factors among Neonates Admitted to Wollega University Referral Hospital Neonatal Intensive Care Unit, East Wollega, Ethiopia. Global Pediatric Health. 2021;8:2333794X211030157. doi: 10.1177/2333794X211030157 34286050 PMC8267021

[93] A. M, T. N, T. E. Perinatal Death and Associated Factors in Wolaita Sodo Referral Hospital, Southern Ethiopia: a Facility Based Cross-Sectional Study. Primary Health Care. 2017.

[94] Mengesha S, Dangisso MH. Burden of stillbirths and associated factors in Yirgalem Hospital, Southern Ethiopia: a facility based cross-sectional study. BMC Pregnancy & Childbirth. 2020;20(1):N.PAG-N.PAG. doi: 10.1186/s12884-020-03296-x . Language: English. Entry Date: In Process. Revision Date: 20210711. Publication Type: journal article. Journal Subset: Biomedical. 33023508 PMC7539424

[95] Tewabe T, Mohammed S, Tilahun Y, Melaku B, Fenta M, Dagnaw T, et al. Clinical outcome and risk factors of neonatal sepsis among neonates in Felege Hiwot referral Hospital, Bahir Dar, Amhara Regional State, North West Ethiopia 2016: a retrospective chart review. BMC research notes. 2017;10(1):265. Epub 2017/07/12. doi: 10.1186/s13104-017-2573-1 . 28693597 PMC5504561

[96] Aliyu S, Yizengaw T, Lemma T. Prevalence and associated factors of uterine rupture during labor among women who delivered in Debre Markos hospital north West Ethiopia. Intern Med. 2016;6(4):1000222.

[97] Getahun WT, Solomon AA, Kassie FY, Kasaye HK, Denekew HT. Uterine rupture among mothers admitted for obstetrics care and associated factors in referral hospitals of Amhara regional state, institution-based cross-sectional study, Northern Ethiopia, 2013–2017. PLoS One. 2018;13(12):e0208470. Epub 2018/12/05. doi: 10.1371/journal.pone.0208470 . 30513120 PMC6279034

[98] Girmay G, Gultie T, Gebremichael G, Afework B, Temesgen G. Determinants of uterine rupture among mothers who gave birth in Jinka and Arba Minch general hospitals, institution-based case–control study, southern Ethiopia, Ethiopia, 2019. Women’s Health. 2020;16:1745506520961722. 10.1177/1745506520961722 PMC7534070 32985385

[99] Bereka TM, Mulat Aweke A, Eshetie Wondie T. Associated Factors and Outcome of Uterine Rupture at Suhul General Hospital, Shire Town, North West Tigray, Ethiopia 2016: A Case-Control Study. Obstetrics and Gynecology International. 2017;2017:8272786. doi: 10.1155/2017/8272786 29403533 PMC5748301

[100] Mengesha MB, Weldegeorges DA, Hailesilassie Y, Werid WM, Weldemariam MG, Welay FT, et al. Determinants of Uterine Rupture and Its Management Outcomes among Mothers Who Gave Birth at Public Hospitals of Tigrai, North Ethiopia: An Unmatched Case Control Study. Journal of pregnancy. 2020;2020:8878037. Epub 2020/11/17. doi: 10.1155/2020/8878037 . 33194231 PMC7641719

[101] Workie A, Getachew Y, Temesgen K, Kumar P JIJRCOG. Determinants of uterine rupture in Dessie Referral Hospital, North East Ethiopia, 2016: case control design. 2018;7(5):1712–7.

[102] Sa A, Tk Y, Tb L, Abdu S. Prevalence and Associated Factors of Uterine Rupture During Labor among Women Who Delivered in Debre Markos Hospital North West Ethiopia. Journal of Internal Medicine. 2020;6:1 to 6.

[103] Neilson J, Lavender T, Quenby S, Wray S. Obstructed labour: Reducing maternal death and disability during pregnancy. British Medical Bulletin. 2003;67(1):191–204. J British Medical Bulletin. doi: 10.1093/bmb/ldg018 14711764

[104] Abasiattai A, Bassey E, Etuk S, Udoma E, Gynaecology. The pattern of obstructed labour in Uyo, South-Eastern Nigeria. Tropical Journal of Obstetrics. 2006;23(2):146–9.

[105] Jeremiah I, Nwagwu V. The pattern of obstructed labour among parturients in a tertiary hospital in southern Nigeria. Port Harcourt Medical Journal. 2012;6(1):89–95.

[106] Ozumba BC, Uchegbu H. Incidence and management of obstructed labour in eastern Nigeria. The Australian & New Zealand journal of obstetrics & gynaecology. 1991;31(3):213–6. Epub 1991/08/01. doi: 10.1111/j.1479-828x.1991.tb02783.x 1804080

[107] Kabakyenga JK, Östergren P-O, Turyakira E, Mukasa PK, Pettersson KO. Individual and health facility factors and the risk for obstructed labour and its adverse outcomes in south-western Uganda. BMC Pregnancy & Childbirth. 2011;11(1):1–10. doi: 10.1186/1471-2393-11-73 21995340 PMC3204267

[108] Tabassum S, Shamsher S, Sadaf R, Rauf B, Begum IJK. Fetomaternal outcome of obstructed labour. 2017;10(3):318.

[109] Zafar S, Taj N, Iqbal R, Masood MS. Maternal outcome in obstructed labour in patients presenting to Nishtar Hospital Multan. The Professional Medical Journal. 2021;28(03):377–81.

[110] Tessema ZT, Teshale AB, Tesema GA, Tamirat KS. Determinants of completing recommended antenatal care utilization in sub-Saharan from 2006 to 2018: evidence from 36 countries using Demographic and Health Surveys. BMC pregnancy and childbirth. 2021;21(1):192. doi: 10.1186/s12884-021-03669-w 33676440 PMC7937261

[111] World Health Organization (WHO). WHO recommendations on antenatal care for a positive pregnancy experience. 2016. 28079998

[112] Heemelaar S, Kabongo L, Ithindi T, Luboya C, Munetsi F, Bauer A-K, et al. Measuring maternal near-miss in a middle-income country: assessing the use of WHO and sub-Saharan Africa maternal near-miss criteria in Namibia. Global Health Action. 2019;12(1):1646036. doi: 10.1080/16549716.2019.1646036 31405363 PMC6713162

[113] Nansubuga E, Ayiga N, Moyer CA. Prevalence of maternal near miss and community-based risk factors in Central Uganda. International journal of gynaecology and obstetrics: the official organ of the International Federation of Gynaecology and Obstetrics. 2016;135(2):214–20. Epub 2016/08/25. doi: 10.1016/j.ijgo.2016.05.009 . 27553504

[114] Tura AK, Trang TL, van den Akker T, van Roosmalen J, Scherjon S, Zwart J, et al. Applicability of the WHO maternal near miss tool in sub-Saharan Africa: a systematic review. BMC pregnancy and childbirth. 2019;19(1):79. doi: 10.1186/s12884-019-2225-7 30808325 PMC6390325

[115] Lawson JB. OBSTRUCTED LABOUR. 1965;72(6):877–80. doi: 10.1111/j.1471-0528.1965.tb01506.x

[116] Ali AA, Adam I. Maternal and perinatal outcomes of obstructed labour in Kassala hospital, Sudan. Journal of obstetrics and gynaecology: the journal of the Institute of Obstetrics and Gynaecology. 2010;30(4):376–7. Epub 2010/05/12. doi: 10.3109/01443611003672096 . 20455721

[117] Rizvi SM, Gandotra N. Maternofetal outcome in obstructed labour in a tertiary care hospital. Int J Reprod Contr Obstet Gynecol. 2017;4:1410–3.

[118] Harrison MS, Ali S, Pasha O, Saleem S, Althabe F, Berrueta M, et al. A prospective population-based study of maternal, fetal, and neonatal outcomes in the setting of prolonged labor, obstructed labor and failure to progress in low- and middle-income countries. Reproductive health. 2015;12 (Suppl 2):S9. Epub 2015/06/13. doi: 10.1186/1742-4755-12-s2-s9 . 26063492 PMC4464213

[119] Khooharo Y, Yousfani JZ, Malik SH, Amber A, Majeed N, Malik NH, et al. Incidence and management of rupture uterus in obstructed labour. Journal of Ayub Medical College, Abbottabad: JAMC. 2013;25(1–2):149–51. Epub 2013/01/01. . 25098081

[120] Mondal S, Chaudhuri A, Kamilya G, Santra D, April-June. Fetomaternal outcome in obstructed labor in a peripheral tertiary care hospital. Medical Journal of Dr DY Patil University. 2013;6(2).

[121] Khan KS, Wojdyla D, Say L, Gülmezoglu AM, Van Look PFA. WHO analysis of causes of maternal death: a systematic review. The Lancet. 2006;367(9516):1066–74. doi: 10.1016/S0140-6736(06)68397-9 16581405

[122] Kidantou HL, Mwampagatwa I, Van Roosmalen J. Uterine rupture: a retrospective analysis of causes, complications and management outcomes at Muhimbili National Hospital in Dar es Salaam, Tanzania. Tanzania journal of health research. 2012;14(3):220–5. Epub 2012/07/01. . 26591760

[123] Pokhrel Ghimire SS. Uterine Rupture: Shifting Paradigm in Etiology. Kathmandu University medical journal (KUMJ). 2018;16(62):146–50. Epub 2019/01/15. . 30636755

[124] World Health Organization (WHO). Managing prolonged and obstructed labour: Education material for teachers of midwifery. Geneva 2008.

[125] Sarkar MK, Halder A, Sciences D. Foeto-Maternal Outcome of Obstructed Labour in a Tertiary Care Hospital of Kolkata. Journal of Evolution of Medical. 2020;9(13):1019–22.

[126] Egziabher TG, Eugene N, Ben K, Fredrick K. Obstetric fistula management and predictors of successful closure among women attending a public tertiary hospital in Rwanda: a retrospective review of records. BMC research notes. 2015;8:774. Epub 2015/12/15. doi: 10.1186/s13104-015-1771-y . 26654111 PMC4676892

[127] Miller S, Lester F, Webster M, Cowan B JJoM, Health Ws. Obstetric fistula: a preventable tragedy. 2005;50(4):286–94. doi: 10.1016/j.jmwh.2005.03.009 15973264

[128] Raassen TJ, Verdaasdonk EG, Vierhout ME. Prospective results after first-time surgery for obstetric fistulas in East African women. International urogynecology journal and pelvic floor dysfunction. 2008;19(1):73–9. Epub 2007/05/12. doi: 10.1007/s00192-007-0389-6 . 17492390

[129] Sachdev PS, Hassan N, Abbasi RM, Das CM. Genito-urinary fistula: a major morbidity in developing countries. Journal of Ayub Medical College, Abbottabad: JAMC. 2009;21(2):8–11. Epub 2009/04/01. . 20524458

[130] Khan S, Roohi M. Obstructed labour: the preventable factors. JPMA The Journal of the Pakistan Medical Association. 1995;45(10):261–3. Epub 1995/10/01. . 8714620

[131] Kiran A, Singh RR, Sinha AN, Srinagar UI. A clinical study of 100 cases of Obstructed Labour and its Fetomaternal Outcome. Journal of Biology Life Science. 2015;6:141–7.

[132] Fasubaa OB, Ezechi OC, Orji EO, Ogunniyi SO, Akindele ST, Loto OM, et al. Delivery of the impacted head of the fetus at caesarean section after prolonged obstructed labour: a randomised comparative study of two methods. Journal of obstetrics and gynaecology: the journal of the Institute of Obstetrics and Gynaecology. 2002;22(4):375–8. Epub 2003/01/11. doi: 10.1080/01443610220141290 . 12521457

[133] Garba B, Sakajiki M, Musa A, Kolawole T, Adeniji A, Adelakun MB. Prevalence and Risk Factors for Perinatal Asphyxia as Seen at a Specialist Hospital in Gusau, Nigeria. 2018.

[134] David AN, Njokanma OF, Iroha E. Incidence of and factors associated with meconium staining of the amniotic fluid in a Nigerian University Teaching Hospital. Journal of Obstetrics and Gynaecology. 2006;26(6):518–20. doi: 10.1080/01443610600797426 17000496

