# Supplementary material for: Obstructed labor and its effect on adverse maternal and fetal outcomes in Ethiopia: A systematic review and meta-analysis
Source: PLoS One. 2022 Sep 30;17(9):e0275400. doi: 10.1371/journal.pone.0275400 (PMC9524671; doi:10.1371/journal.pone.0275400)
Supplement: S1 Checklist — (DOCX) [file pone.0275400.s001.docx]

**Identification of studies via other methods**

**Identification of studies via databases and registers**

Records identified from:

Websites (n =100)

Organisations (n=0)

Citation searching (n = 34)

etc.

Records removed *before screening*:

Duplicate records removed (n = 400)

Records marked as ineligible by automation tools (n = 0)

Records removed for other reasons (n = 0)

Records identified from*:

Databases (n = 1,471)

Registers (n=0 )

**Identification**

Records screened

(n =1071)

Records excluded**

(n = 901)

Reports not retrieved

(n =104)

Reports sought for retrieval

(n = 30)

Reports sought for retrieval

(n =150 )

Reports not retrieved

(n = 751)

**Screening**

Reports assessed for eligibility

(n = 20)

Reports excluded:

Reason 1 (n =18 exposure of interest not reported)

Reports assessed for eligibility

(n = 150)

Reports excluded:

Reason 1 (n =62 exposure of interest not reported)

Reason 2 (n =2 commentary)

Reason 3 (n = 1 review)

etc.

Studies included in review

(n =87)

Reports of included studies

(n =87)

**Included**

*Consider, if feasible to do so, reporting the number of records identified from each database or register searched (rather than the total number across all databases/registers).

**If automation tools were used, indicate how many records were excluded by a human and how many were excluded by automation tools.

*From:*  Page MJ, McKenzie JE, Bossuyt PM, Boutron I, Hoffmann TC, Mulrow CD, et al. The PRISMA 2020 statement: an updated guideline for reporting systematic reviews. BMJ 2021;372:n71. doi: 10.1136/bmj.n71. For more information, visit: <http://www.prisma-statement.org/>
