# Supplementary material for: Obstructed labor and its effect on adverse maternal and fetal outcomes in Ethiopia: A systematic review and meta-analysis
Source: PLoS One. 2022 Sep 30;17(9):e0275400. doi: 10.1371/journal.pone.0275400 (PMC9524671; doi:10.1371/journal.pone.0275400)
Supplement: S1 File — (DOC) [file pone.0275400.s002.doc]

**Supplementary file 2: search strategy used for the systematic and Meta-analysis on obstructed labour and its effect on adverse maternal and fetal outcomes in Ethiopia**

(((((((((((((((("Obstructed labour"[All Fields] OR ("dystocia"[MeSH Terms] OR "dystocia"[All Fields] OR ("abnormal"[All Fields] AND "labour"[All Fields]) OR "abnormal labour"[All Fields])) OR ("dystocia"[MeSH Terms] OR "dystocia"[All Fields] OR ("labour"[All Fields] AND "dystocia"[All Fields]) OR "labour dystocia"[All Fields])) OR "Labour abnormality"[All Fields]) OR ("maternal mortality"[MeSH Terms] OR ("maternal"[All Fields] AND "mortality"[All Fields]) OR "maternal mortality"[All Fields])) OR ("labour complication"[All Fields] OR "obstetric labor complications"[MeSH Terms] OR ("obstetric"[All Fields] AND "labor"[All Fields] AND "complications"[All Fields]) OR "obstetric labor complications"[All Fields] OR ("labor"[All Fields] AND "complication"[All Fields]) OR "labor complication"[All Fields])) OR "Maternal near-miss"[All Fields]) OR "Neonatal near miss"[All Fields]) OR ("perinatal mortality"[MeSH Terms] OR ("perinatal"[All Fields] AND "mortality"[All Fields]) OR "perinatal mortality"[All Fields] OR "perinatal death"[MeSH Terms] OR ("perinatal"[All Fields] AND "death"[All Fields]) OR "perinatal death"[All Fields] OR ("perinatal"[All Fields] AND "mortality"[All Fields]))) OR ("fistula"[MeSH Terms] OR "fistula"[All Fields])) OR ("caesarean section"[All Fields] OR "cesarean section"[MeSH Terms] OR ("cesarean"[All Fields] AND "section"[All Fields]) OR "cesarean section"[All Fields])) OR ("uterine rupture"[MeSH Terms] OR ("uterine"[All Fields] AND "rupture"[All Fields]) OR "uterine rupture"[All Fields])) OR "Instrumental delivery"[All Fields]) OR "Operative delivery"[All Fields]) OR "Prolonged labor"[All Fields]) OR "Adverse birth outcome"[All Fields]) AND ((((("maternal death"[MeSH Terms] OR ("maternal"[All Fields] AND "death"[All Fields]) OR "maternal death"[All Fields]) OR "Adverse maternal outcome"[All Fields]) OR ("perinatal death"[MeSH Terms] OR ("perinatal"[All Fields] AND "death"[All Fields]) OR "perinatal death"[All Fields])) OR "Adverse perinatal outcome"[All Fields]) OR "Feto-maternal outcomes"[All Fields])) AND ("ethiopia"[MeSH Terms] OR "ethiopia"[All Fields])

| **Result:** |
| --- |
| **5870** |
| **Translations:** |
| | Abnormal labour | "dystocia"[MeSH Terms] OR "dystocia"[All Fields] OR ("abnormal"[All Fields] AND "labour"[All Fields]) OR "abnormal labour"[All Fields] | | --- | --- | | Labour dystocia | "dystocia"[MeSH Terms] OR "dystocia"[All Fields] OR ("labour"[All Fields] AND "dystocia"[All Fields]) OR "labour dystocia"[All Fields] | | Maternal mortality | "maternal mortality"[MeSH Terms] OR ("maternal"[All Fields] AND "mortality"[All Fields]) OR "maternal mortality"[All Fields] | | Labour complication | "labour complication"[All Fields] OR "obstetric labor complications"[MeSH Terms] OR ("obstetric"[All Fields] AND "labor"[All Fields] AND "complications"[All Fields]) OR "obstetric labor complications"[All Fields] OR ("labor"[All Fields] AND "complication"[All Fields]) OR "labor complication"[All Fields] | | perinatal mortality | "perinatal mortality"[MeSH Terms] OR ("perinatal"[All Fields] AND "mortality"[All Fields]) OR "perinatal mortality"[All Fields] OR "perinatal death"[MeSH Terms] OR ("perinatal"[All Fields] AND "death"[All Fields]) OR "perinatal death"[All Fields] OR ("perinatal"[All Fields] AND "mortality"[All Fields]) | | Fistula | "fistula"[MeSH Terms] OR "fistula"[All Fields] | | Cesarean section | "caesarean section"[All Fields] OR "cesarean section"[MeSH Terms] OR ("cesarean"[All Fields] AND "section"[All Fields]) OR "cesarean section"[All Fields] | | uterine rupture | "uterine rupture"[MeSH Terms] OR ("uterine"[All Fields] AND "rupture"[All Fields]) OR "uterine rupture"[All Fields] | | Maternal death | "maternal death"[MeSH Terms] OR ("maternal"[All Fields] AND "death"[All Fields]) OR "maternal death"[All Fields] | | Perinatal death | "perinatal death"[MeSH Terms] OR ("perinatal"[All Fields] AND "death"[All Fields]) OR "perinatal death"[All Fields] | | Ethiopia | "ethiopia"[MeSH Terms] OR "ethiopia"[All Fields] | |
| **Database:** |
| PMC |
