# Supplementary material for: Obstructed labor and its effect on adverse maternal and fetal outcomes in Ethiopia: A systematic review and meta-analysis
Source: PLoS One. 2022 Sep 30;17(9):e0275400. doi: 10.1371/journal.pone.0275400 (PMC9524671; doi:10.1371/journal.pone.0275400)
Supplement: S2 File — (DOC) [file pone.0275400.s003.doc]

| **Effect of Obstructed labour on perinatal outcome in Ethiopia: A systematic review and Meta-Analysis** | | | | | | | | |
| --- | --- | --- | --- | --- | --- | --- | --- | --- |
| S no | **Author name** | **Study setting** | **Study design** | **Region** | **Year** | **Study Population** | **Sample size** | **Outcome reported** |
|  | Abdo, A. A. et al | Facility based | Cross sectional | SNNPR | 2020 | Postpartum mothers | 4004 | Caesarean section |
|  | Abdo, R. A et al | Facility based | Cross sectional | SNNPR | 2019 | Postpartum mothers +Card review | 279 | Birth asphyxia |
|  | Abera, Kebebush | Facility based | Cross sectional | Addis Abeba | 2014 | Postpartum mothers | 160 | Postpartum hemorrhage |
|  | Abera, T et al | Facility based | Cross sectional | Oromia | 2021 | Neonates | 312 | Neonatal mortality |
|  | Abraham, W. & Berhan, Y. | Facility based | Case control | SNNPR | 2014 | Laboring mother | 844 | Labor abnormalities |
|  | Addisu et al | Facility based | Cross sectional | Amhara | 2018 | Postpartum mothers | 495 | meconium stained amniotic fluid |
|  | Ahmed et al. | Facility based | Cross sectional | Amhara | 2018 | Women who had uterine rupture | 376 | Uterine rupture |
|  | Alebachew Bayih et al. | Facility based | Cross sectional | Harrari | 2019 | Newborns | 403 | Neonatal hypothermia |
|  | Aliyu et al | Facility based | Cross sectional | Amhara | 2016 | Postpartum mothers | 880 | Uterine rupture |
|  | Aragaw YA | Facility based | Cross sectional | Oromia | 2016 | Postpartum mothers | 3786 | Perinatal mortality |
|  | Asaye M M | Facility based | Cross sectional | Amhara | 2020 | All pregnant women who were in labor, delivered or aborted, or within 42 days of postpartum period | 303 | Maternal near miss |
|  | Asefa et al | Facility based | Retrospective review | Oromia | 2016 | Postpartum mothers | 9585 | Perinatal mortality |
|  | Astatikie et al | Facility based | Cross sectional | Amhara | 2017 | Women who had uterine rupture | 242 | Uterine rupture |
|  | Ayalew et al | Facility based | Cross sectional | Amhara | 2020 | Postpartum mothers | 433 | Caesarean section |
|  | Ayano, B & Guto, A | Facility based | Retrospective review | Addis Abeba | 2018 | Women who had CS | 582 | Indications and Outcomes of Emergency Caesarean Section |
|  | A. Browning, S. Whiteside | Facility based | Retrospective review | Amhara | 2015 | Women with genital tract fistulas | 1057 | Outcomes of repair of rectovaginal fistula |
|  | Dessalegn F N et al | Facility based | Case control | Oromia | 2020 | All pregnant women who were in labor, delivered or aborted, or within 42 days of postpartum period | 321 | Maternal near miss |
|  | Eshete A et al | Facility based | Retrospective review | SNNPR | 2018 | Postpartum mothers | 2,498 | Uterine rupture and fetomaternal outcome |
|  | Abebe et al. | Facility based | Retrospective review | Amhara | 2016 | Postpartum mothers | 2,967 | Cesarean section |
|  | Fesseha et al | Facility based | Retrospective review | National review | 2011 | Postpartum mothers | 797 facilities | Cesarean section |
|  | Fikre et al. | Facility based | Case control | SNNPR | 2021 | Postpartum mothers | 424 | Still birth |
|  | Gdiom Gebreheat et al. | Facility based | Cross sectional | Tigray | 2018 | Neonates | 422 | Perinatal Asphyxia |
|  | Gebrehiwot, B.T. Tewolde | Facility based | Retrospective review | National review | 2014 | All cases of maternal death and near miss | 2774 | Maternal death & Maternal near miss |
|  | Gebretsadik et al | Facility based | Cross sectional | SNNPR | 2020 | Women who had uterine rupture | 331 | Uterine rupture |
|  | Gebretsadik et al | Facility based | Retrospective review | SNNPR | 2020 | Patients who died after being admitted and receiving obstetric and gynecologic care. | 77 | Maternal mortality |
|  | Geleto et al. | Facility based | Retrospective review | National review | 2020 | All public and private health facilities, which offered EmONC services | 293 hospitals | Cesarean section |
|  | Geleto et al. | Facility based | Cross sectional | National review | 2020 | All public and private health facilities | All public and private health facilities | Obstetric case fatality |
|  | Getachew A et al | Facility based | Cross sectional | Oromia | 2021 | All records of mothers presented with obstructed labor | 277 | Feto-Maternal Outcomes of Obstructed Labor |
|  | Getahun T W et al | Facility based | Cross sectional | Amhara | 2018 | Mothers who had taken obstetrics care within the third trimester oftheir pregnancy | 756 | Uterine rupture |
|  | Gidey et al. | Facility based | Case control | Tigray | 2013 | All the charts of mother’s who died due to pregnancy related complication during pregnancy, labor & delivery and up to 42 days after delivery | 310 | Maternal mortality |
|  | Girmay G et al | Facility based | Case control | SNNPR | 2020 | Women diagnosed with uterine rupture | 448 | Uterine rupture |
|  | Goba G et al | Facility based | Case control | Tigray | 2017 | Postpartum mothers | 378 | Perinatal mortality |
|  | Habitamu et al | Facility based | Cross sectional | Amhara | 2019 | Postpartum mothers | 144 | Postpartum hemorrhage |
|  | Hailemariam H A et al | Facility based | Cross sectional | Addis Abeba | 2020 | Postpartum mothers | 248 | Meconium stained amniotic fluid |
|  | Kassahun E A et al | Facility based | Retrospective cross-sectional | Amhara | 2020 | Postpartum mothers | 364 | Nonreassuring Fetal Heart Rate |
|  | Kebede BA et al | Facility based | Cross sectional | SNNPR | 2019 | Postpartum mothers | 422 | Postpartum hemorrhage |
|  | Kumela L et al | Facility based | Case control | Oromia | 2020 | Postpartum mothers | 183 | Maternal near miss |
|  | Legesse T et al | Facility based | Case control | Oromia | 2017 | Postpartum mothers | 600 | Maternal mortality |
|  | Lindtjørn B et al | Facility based | Implementation study | SNNPR | 2017 | 66 Facilities in southern Ethiopia | 66 Facilities | Maternal death |
|  | Liyew E F et al | Facility based | Cross sectional | Addis Abeba | 2017 | All pregnant women who were in labor, delivered or aborted, or within 42 days of postpartum period | 29,697 | Maternal near miss |
|  | Mache G A et al | Facility based | Cross sectional | SNNPR | 2021 | Postpartum mothers | 300 | Cesarean section |
|  | Bereka T M et al | Facility based | Case control | Tigray | 2017 | Postpartum mothers | 336 | Uterine rupture |
|  | Mekango DE et al | Facility based | Case control | Tigray | 2017 | All pregnant women who were in labor, delivered or aborted, or within 42 days of postpartum period | 308 | Maternal near miss |
|  | Melesse M B et al | Facility based | Comparative cross-sectional study | Amhara | 2020 | Postpartum mothers | 724 | Cesarean section |
|  | Mengesha et al. | Facility based | Prospective cohort | Tigray | 2016 | All mothers who gave a live birth | 1024 | Neonatal mortality |
|  | Mengesha M B et al | Facility based | Cross sectional | Tigray | 2019 | Mothers who gave birth by caesarean delivery | 338 | Maternal and fetal outcomes of cesarean delivery |
|  | Mengesha M B et al | Facility based | Case control | Tigray | 2020 | Postpartum mothers | 405 | Uterine rupture |
|  | Mengesha and Dangisso | Facility based | Cross sectional | SNNPR | 2020 | Postpartum mothers | 374 | Still birth |
|  | Mengistie et al | Facility based | Cross sectional | SNNPR | 2016 | Women diagnosed with uterine rupture | 115 | Maternal and Perinatal Outcomes of Uterine Rupture |
|  | Moges A et al | Facility based | Retrospective cross-sectional | SNNPR | 2015 | Cesarean deliveries | 281 | Cesarean section |
|  | Muleta et al | Facility based | Retrospective cross-sectional | National review | 2010 | Women with obstetric fistula | 14,928 | Obstetric fistula |
|  | Roro et al | Community based | Case control | Oromia | 2018 | All perinatal deaths | 219 | Perinatal mortality |
|  | Sidamo N B et al | Facility based | Cross sectional | SNNPR | 2019 | Women admitted for delivery | 346 | Poor Apgar Score |
|  | Abayneh Aklilu Solomon | Facility based | Retrospective cross-sectional | Amhara | 2019 | Laboring mothers | 323 | Cesarean section |
|  | Tadesse, E and Worku B | Facility based | Retrospective cross-sectional | Addis Abeba | 2003 | All deliveries included were between 28 weeks and 42 weeks of GA | 8986 | Perinatal mortality |
|  | Tasew H et al | Facility based | Case control | Tigray | 2019 | Women admitted for delivery | 315 | Still birth |
|  | Tasew et al | Facility based | Case control | Tigray | 2018 | All deliveries mothes | 264 | Birth asphyxia |
|  | Tesfaye S et al | Facility based | Case control | SNNPR | 2019 | Still births or early neonatal deaths | 821 | Perinatal mortality |
|  | Tessema et al | Facility based | Data base review | National review | 2017 | GBD data | 15234 | Maternal mortality |
|  | Tewabe et al | Facility based | Retrospective chart review | Amhara | 2017 | Neonates | 225 | Neonatal sepsis |
|  | Tura A K et al | Facility based | Cross sectional | Harrari | 2018 | All women who underwent CS | 980 | Cesarean section |
|  | Wayessa J Z | Facility based | Cross sectional | Oromia | 2018 | All newborns delivered | 371 | Birth asphyxia |
|  | Welegebriel et al | Facility based | Case control | SNNPR | 2019 | Mothers registered in the hospitals for maternal health service | 547 | Still birth |
|  | Wonde and Mihretie | Facility based | Retrospective review | Tigray | 2019 | Postpartum mothers | 91 | Maternofetal outcomes of obstructed labor |
|  | Wondie et al. | Facility based | Cross sectional | Amhara | 2019 | Women who delivered | 520 | Cesarean section |
|  | Workie A et al | Facility based | Case control | Amhara | 2018 | Women who delivered | 210 | Uterine rupture |
|  | Wosenu L et al | Facility based | Case control | Amhara | 2018 | All live newborns after 28 weeks GA | 273 | Birth asphyxia |
|  | Yaya Y et al | Community based | Follow up | SNNPR | 2015 | Birth registry of pregnant mothers | 10,987 | Maternal mortality |
|  | Yirgu R et al | Community based | Nested Case control | Amhara | 2016 | Pregnant mothers | 306 | Perinatal mortality |
|  | Mihiretu A. et al | Facility based | Cross sectional | SNNPR | 2017 | Postpartum mother | 300 | Perinatal death |
|  | Belay,HG. et al | Facility based | Cross sectional | Amhara | 2019 | Neonates | 404 | Neonatal near miss |
|  | Berhe Y Z et al | Facility based | Case control | Tigray | 2020 | Neonates | 390 | Birth asphyxia |
|  | Fantu S et al | Facility based | Cross sectional | Oromia | 2010 | Postpartum mother | 179 | Multiple outcomes |
|  | Gendisha, G et al | Facility based | Cross sectional | Tigray | 2017 | Postpartum mother | 660 | Multiple outcomes |
|  | Gessessew A and Mesfin M | Facility based | Retrospective review | Tigray | 2003 | Postpartum mother | 191 | Multiple outcomes |
|  | Halil, H et al | Facility based | Cross sectional | SNNPR | 2020 | Postpartum mother | 300 | Cesarean section |
|  | Kahsay, S. et al | Facility based | Case control | Tigray | 2015 | Postpartum mother | 456 | Cesarean section |
|  | Mengistie, A et al | Facility based | Cross sectional | Amhara | 2019 | Postpartum mother | 310 | Still birth |
|  | Mulugeta, T et al | Facility based | Case control | Addis Abeba | 2020 | Asphyxiated newborns | 213 | Perinatal asphyxia |
|  | Sa, Aliyu et al | Facility based | Cross sectional | Amhara | 2020 | Postpartum mother | 880 | Uterine rupture |
|  | Tenaw, Z et al | Facility based | Cross sectional | SNNPR | 2020 | Postpartum mother | 514 | Cesarean section |
|  | Mesfin, S et al 2021 | Facility based | Cross sectional | Harrari | 2021 | Postpartum mother | 653 | Postpartum hemorrhage |
|  | Tatek, A et al | Facility based | Cross sectional | Amhara | 2014 | Postpartum mother | 228 | Postpartum hemorrhage |
|  | Gedefaw G et al | Survey | Cross sectional | National | 2021 | Women aged 15–49 years | 7590 | Obstetric fistula |
|  | Dereje, B and Abebe, E | Facility based | Cross sectional | Oromia | 2019 | Women with obstetric fistula | 62 | Obstetric fistula |
|  | Bayou, G and Berhan, Y | Facility based | Case control | SNNPR | 2012 | stillbirths and neonatal deaths | 1356 | Perinatal mortality |
|  | Abdulrazaq B et al | Facility based | Case control | Oromia | 2020 | Records of Maternal near-miss | 344 | Maternal near-miss |
