# Supplementary material for: Obstructed labor and its effect on adverse maternal and fetal outcomes in Ethiopia: A systematic review and meta-analysis
Source: PLoS One. 2022 Sep 30;17(9):e0275400. doi: 10.1371/journal.pone.0275400 (PMC9524671; doi:10.1371/journal.pone.0275400)
Supplement: S3 File — (DOC) [file pone.0275400.s004.doc]

**Supplemental material 4. Quality assessment of studies included in this systematic review and meta-analysis on the effect of obstructed labour on perinatal outcome in Ethiopia: A systematic review and Meta-Analysis**

| **Prospective studies** | Representativeness of the exposed cohort | Selection of the non-exposed cohort | Ascertainment of exposure | Demonstration that outcome of interest was not present at the start of the study | Study controls for most important factor | Study controls for an additional factor | Assessment of outcome | Was follow-up long enough for outcomes to occur (>7years) | Adequacy of follow up of cohorts (loss-to-follow-up <20%) | **Total score** |
| --- | --- | --- | --- | --- | --- | --- | --- | --- | --- | --- |
| Mengesha et al. 2016 | * | * | * | * | * | - | * | * | * | 8 |
| Lindtjørn B et al 2017 | * | - | * | * | - | - | * | * | * | 6 |
| **Case-control studies** | Adequate case definition | Representativeness of the cases | Selection of Controls | Definition of Controls | Study controls for most important factor | Study controls for an additional factor | Ascertainment of exposure | The same method of ascertainment for cases and controls | the same non-response rate for both groups | **Total score** |
| Abraham, W. & Berhan, Y. 2014 | * | * | * | * | * | * | * | * | * | 9 |
| Dessalegn F N et al 2020 | * | * | * | * | * | * | * | * | * | 8 |
| Fikre et al. 2021 | * | * | * | * | - | * | * | * | * | 8 |
| Gidey et al. 2013 | * | * | * | * | * | * | * | * | * | 8 |
| Girmay G et al 2020 | - | * | * | * | * | * | * | * | * | 8 |
| Goba G et al 2017 | - | * | * | * | * | * | - | * | * | 7 |
| Kumela L et al 2020 | * | * | * | * | - | - | * | * | * | 7 |
| Legesse T et al 2017 | * | * | * | * | * | - | * | * | * | 8 |
| Bereka T M et al 2017 | - | * | * | * | - | * | * | * | * | 7 |
| Mekango DE et al 2017 | * | * | * | * | * | * | * | * | * | 9 |
| Mengesha M B et al 2020 | * | * | * | * | * | * | * | * | * | 9 |
| Roro et al 2017 | * | * | * | * | * | * | * | * | * | 9 |
| Tasew H et al 2019 | * | * | * | * | - | * | * | * | * | 8 |
| Tasew et al 2018 | * | * | * | * | - | * | * | * | * | 8 |
| Tesfaye S et al 2019 | * | * | * | * | * | * | - | - | * | 7 |
| Welegebriel et al 2019 | * | * | * | * | * | * | * | * | * | 9 |
| Workie A et al 2019 | * | - | * | * | - | * | * | * | * | 7 |
| Wosenu L et al 2019 | * | * | * | * | * | * | * | * | * | 9 |
| Yirgu R et al 2016 | * | * | * | * | * | * | * | * | * | 9 |
| Berhe Y Z et al | - | * | * | * | - | * | - | * | * | 6 |
| Kahsay, S. et al | - | * | * | * | * | * | * | * | * | 8 |
| Mulugeta, T et al | * | * | * | * | * | * | - | * | * | 8 |
| Bayou, G and Berhan, Y 2012 | * | * | * | * | * | * | * | * | * | 9 |
| Abdulrazaq B et al, 2020 | * | * | * | * | * | * | * | * | * | 9 |
| **Cross-sectional studies** | Representativeness of the sample | Sample size | The response rate | Ascertainment of the exposure(Risk factor) | Study controls for most important factor | Study controls for an additional factor | Assessment of the outcome | Statistical test |  | **Total score** |
| Abdo, A. A. et al 2020 | * | * | * | * | - | - | ** | * |  | 7 |
| Abdo, R. A et al 2019 | * | * | * | * | * | * | ** | * |  | 9 |
| Abebe, F et al 2018 | * | * | * | * | * | * | ** | * |  | 9 |
| Abera, Kebebush 2014 | * | * | * | * | - | - | * | - |  | 5 |
| Abera, T et al 2021 | * | * | * | * | - | * | ** | * |  | 8 |
| Addisu et al 2018 | * | * | * | * | * | * | ** | * |  | 9 |
| Ahmed et al. 2018 | * | * | * | * | - | * | ** | * |  | 8 |
| Alebachew Bayih et al. 2019 | * | * | * | ** | - | * | ** | * |  | 9 |
| Aliyu et al 2016 | * | * | * | * | * | * | * | * |  | 8 |
| Aragaw YA 2016 | * | * | * | * | - | * | * | * |  | 7 |
| Asaye M M 2020 | * | * | * | ** | - | * | ** | * |  | 9 |
| Asefa et al 2016 | * | - | * | * | - | - | * | * |  | 5 |
| Astatikie et al 2017 | * | * | * | * | * | - | ** | * |  | 8 |
| Ayalew et al 2020 | * | * | * | * | - | * | ** | * |  | 8 |
| Ayano, B & Guto, A 2018 | - | - |  | * | - | * | * | * |  | 4 |
| Browning, S. Whiteside 2015 | * | * | * | * | - | - | ** | * |  | 7 |
| Degno et al. 2021 | * | * | * | * | - | * | * | * |  | 7 |
| Eshete A et al 2018 | - | - | * | * | - | * | ** | * |  | 6 |
| Abebe et al. 2016 | * | * | * | * | * | * | ** | * |  | 9 |
| Fesseha et al 2011 | * | * | * | * | - | - | ** | * |  | 7 |
| Gdiom Gebreheat et al. 2018 | * | * | * | * | * | * | ** | * |  | 9 |
| Gebrehiwot, B.T. Tewolde 2014 | * | * | * | * | - | - | ** | * |  | 7 |
| Gebretsadik et al 2020  (Uterine R) | * | * | * | ** | - | * | ** | * |  | 9 |
| Gebretsadik et al 2020 (maternal death) | * | * | * | * | - | - | ** | * |  | 7 |
| Geleto et al. 2020  (MM & CS) | * | * | * | * | * | * | ** | * |  | 9 |
| Geleto et al. 2020  (Obstetric case fatality) | * | * | * | ** | * | * | * | * |  | 9 |
| Getachew A et al 2021 | * | * | * | * | * | * | ** | * |  | 9 |
| Getahun T W et al 2018 | * | * | * | * | * | * | ** | * |  | 9 |
| Habitamu et al 2019 | * | * | * | * | * | * | * | * |  | 8 |
| Hailemariam H A et al 2020 | * | * | * | * | - | * | ** | * |  | 8 |
| Kebede BA et al 2019 | * | * | * | * | * | * | ** | * |  | 9 |
| Liyew E F et al 2017 | * | * | * | * | - | - | ** | * |  | 7 |
| Mache G A et al 2021 | - | * | * | * | * | * | * | * |  | 7 |
| Melesse M B et al 2020 | * | * | * | * | * | * | ** | * |  | 9 |
| Mengesha M B et al 2019 | * | * | * | * | - | * | * | * |  | 7 |
| Mengesha and Dangisso 2020 | * | * | * | * | - | * | * | * |  | 7 |
| Mengistie et al 2016 | - | - | - | * | - | * | ** | * |  | 5 |
| Moges A et al 2015 | * | * | * | * | - | - | * | * |  | 6 |
| Muleta et al 2010 | * | * | * | * | * | - | ** | * |  | 8 |
| Sidamo N B et al 2019 | * | * | * | * | - | * | ** | * |  | 8 |
| Abayneh Aklilu Solomon 2019 | * | * | * | * | - | * | * | * |  | 7 |
| Tadesse, E and Worku B 2003 | * | * | * | * | - | - | * | * |  | 6 |
| Tessema et al 2017 | * | * | * | ** | - | - | ** | * |  | 8 |
| Tewabe et al 2017 | * | * | * | * | - | * | * | * |  | 8 |
| Tura A K et al 2018 | * | * | * | ** | - | - | ** | * |  | 8 |
| Wayessa J Z 2018 | * | * | * | * | - | * | * | * |  | 7 |
| Wonde and Mihretie 2019 | * | * | * | * | - | - | ** | * |  | 7 |
| Wondie et al. 2019 | * | * | * | * | - | * | ** | * |  | 8 |
| Yaya Y et al 2015 | * | * | * | ** | - | - | ** | * |  | 8 |
| Mihiretu A. et al 2017 | * | * | * | * | * | * | ** | * |  | 9 |
| Belay,HG. et al 2019 | * | * | * | * | - | * | ** | * |  | 8 |
| Fantu S et al 2010 | * | * | * | * | * | * | ** | * |  | 9 |
| Gendisha, G et al 2017 | * | * | * | * | - | - | * | * |  | 6 |
| Gessessew A and Mesfin M 2003 | * | * | * | ** | - | - | ** | - |  | 7 |
| Halil, H et al 2020 | * | * | * | * | * | * | ** | * |  | 9 |
| Mengistie, A et al 2019 | * | * | * | * | * | * | ** | * |  | 9 |
| Sa, Aliyu et al 2020 | * | * | * | * | * | * | ** | * |  | 9 |
| Tenaw, Z et al 2020 | * | * | * | * | * | * | * | * |  | 8 |
| Yimer, N et al 2020 | * | * | * | * | * | * | * | * |  | 8 |
| Mesfin, S et al 2021 | * | * | * | * | * | * | ** | * |  | 9 |
| Tatek, A et al 2014 | * | * | * | * | - | * | * | * |  | 7 |
| Dereje, B and Abebe, E 2019 | * | * | * | * | - | - | ** | - |  | 6 |
| Gedefaw G et al | * | * | * | * | * | * | ** | * |  | 9 |

According to the Newcastle-Ottawa Scale (NOS) criteria [1]

NA: Not Applicable

1. Wells G, Shea B, O'Connell D, Peterson j, Welch V, Losos M, et al. The Newcastle–Ottawa Scale (NOS) for Assessing the Quality of Non-Randomized Studies in Meta-Analysis. 2000;ᅟ
